# Modelling visibility judgments using models of decision confidence

**DOI:** 10.3758/s13414-021-02284-3

**Published:** 2021-06-04

**Authors:** Manuel Rausch, Sebastian Hellmann, Michael Zehetleitner

**Affiliations:** Philosophisch-pädagogische Fakultät, Fachgebiet Psychologie II, Catholic University Eichstätt-Ingolstadt, 85072 Eichstätt, Germany

**Keywords:** Visual awareness, Cognitive modelling, Visibility, Consciousness, Metacognition

## Abstract

How can we explain the regularities in subjective reports of human observers about their subjective visual experience of a stimulus? The present study tests whether a recent model of confidence in perceptual decisions, the weighted evidence and visibility model, can be generalized from confidence to subjective visibility. In a postmasked orientation identification task, observers reported the subjective visibility of the stimulus after each single identification response. Cognitive modelling revealed that the weighted evidence and visibility model provided a superior fit to the data compared with the standard signal detection model, the signal detection model with unsystematic noise superimposed on ratings, the postdecisional accumulation model, the two-channel model, the response-congruent evidence model, the two-dimensional Bayesian model, and the constant noise and decay model. A comparison between subjective visibility and decisional confidence revealed that visibility relied more on the strength of sensory evidence about features of the stimulus irrelevant to the identification judgment and less on evidence for the identification judgment. It is argued that at least two types of evidence are required to account for subjective visibility, one related to the identification judgment, and one related to the strength of stimulation.

**Supplementary Information:**

The online version contains supplementary material available at 10.3758/s13414-021-02284-3.

## Introduction

 Observers’ subjective reports about their conscious experiences are often considered to be problematic or even unsuitable for objective science (Eriksen, 1960; Hannula et al., 2005; Irvine, 2012; Schmidt & Vorberg, 2006). Nevertheless, a complete science of psychology should be able to explain all human behavior, including participants’ utterances about their conscious experience (Dennett, 2003, 2007). One of the most useful tools to identify regularities underlying human behavior is cognitive modelling (e.g., McClelland, 2009). Once identified, these regularities demand a scientific explanation. In the present study, we use cognitive modelling to identify patterns in observers’ reports about the degree to which they are consciously seeing a stimulus (i.e., the subjective visibility). For this purpose, we test whether previously proposed models of confidence in binary perceptual choices, including the *weighted evidence and visibility model* (Rausch et al., 2018) can be used to account for visibility judgments as well.

### Subjective visibility versus decisional confidence

Applying previously established models of decisional confidence to visibility judgments is a natural place to approach the problem of modelling visibility. The reason is that many different models of confidence already exist (Aitchison et al., 2015; Green & Swets, 1966; Maniscalco et al., 2016; Maniscalco & Lau, 2016; Moran et al., 2015; Pleskac & Busemeyer, 2010; Ratcliff & Starns, 2009, 2013; Rausch et al., 2018; Rausch & Zehetleitner, 2017), and visibility judgments and confidence are often thought to be closely related, or even interchangeable (e.g., Lau & Rosenthal, 2011; Seth et al., 2008). However, it should not be taken for granted that models developed for decisional confidence can be applied to visibility judgments, as some important differences between confidence and visibility judgments exist: In a series of psychophysical experiments, the majority of observers reported confidence that the response is correct at a level of stimulation where they not yet reported seeing the stimulus; only when the stimulation was stronger, they would report a visual experience in addition to confidence in being correct (Rausch & Zehetleitner, 2016; Zehetleitner & Rausch, 2013). Moreoever, confidence and error monitoring judgments were sensitive to errors in a discrimination task even when observers report not seeing the stimulus (Charles et al., 2013; Jachs et al., 2015). Extreme dissociations between subjective visibility and confidence have been reported with so-called blindsight patients: After lesions to primary visual cortex, these patients report to be blind in the visual field contralateral to the impaired brain area, although they are able to discriminate visual stimuli presented in their seemingly blind visual field in forced-choice tasks with remarkable accuracy (Weiskrantz, 1986). Some blindsight patients report a considerable degree of confidence that judgments about a stimulus presented in their blind hemifield are correct (Sahraie et al., 1998), and wager the same amount of money on judgments on stimuli in the blind as in the intact hemifield when performance is balanced (Persaud et al., 2007). In addition, confidence and visibility judgments are sometimes differentially related to task accuracy. In a masked orientation task, a masked shape discrimination task, low contrast orientation tasks, and random dot motion identification task, confidence was more closely associated with task performance than subjective visibility (Rausch et al., 2015; Rausch & Zehetleitner, 2016), whereas the reverse relationship was observed in a masked object identification task (Sandberg et al., 2010) and a discrimination task about masked face expressions (Wierzchoń et al., 2014). Finally, visibility is closely correlated with Type 1 sensitivity, which quantifies observers’ ability to discriminate the stimulus, but visibility is only weakly predictive of Type 2 sensitivity, i.e., the degree to which confidence judgments differentiate between correct and incorrect discrimination judgments (Jachs et al., 2015). Taken together, these studies indicate that it cannot be assumed a priori that models of confidence can also be used to account for visibility judgments.

### The weighted evidence and visibility model

**Fig. 1:**
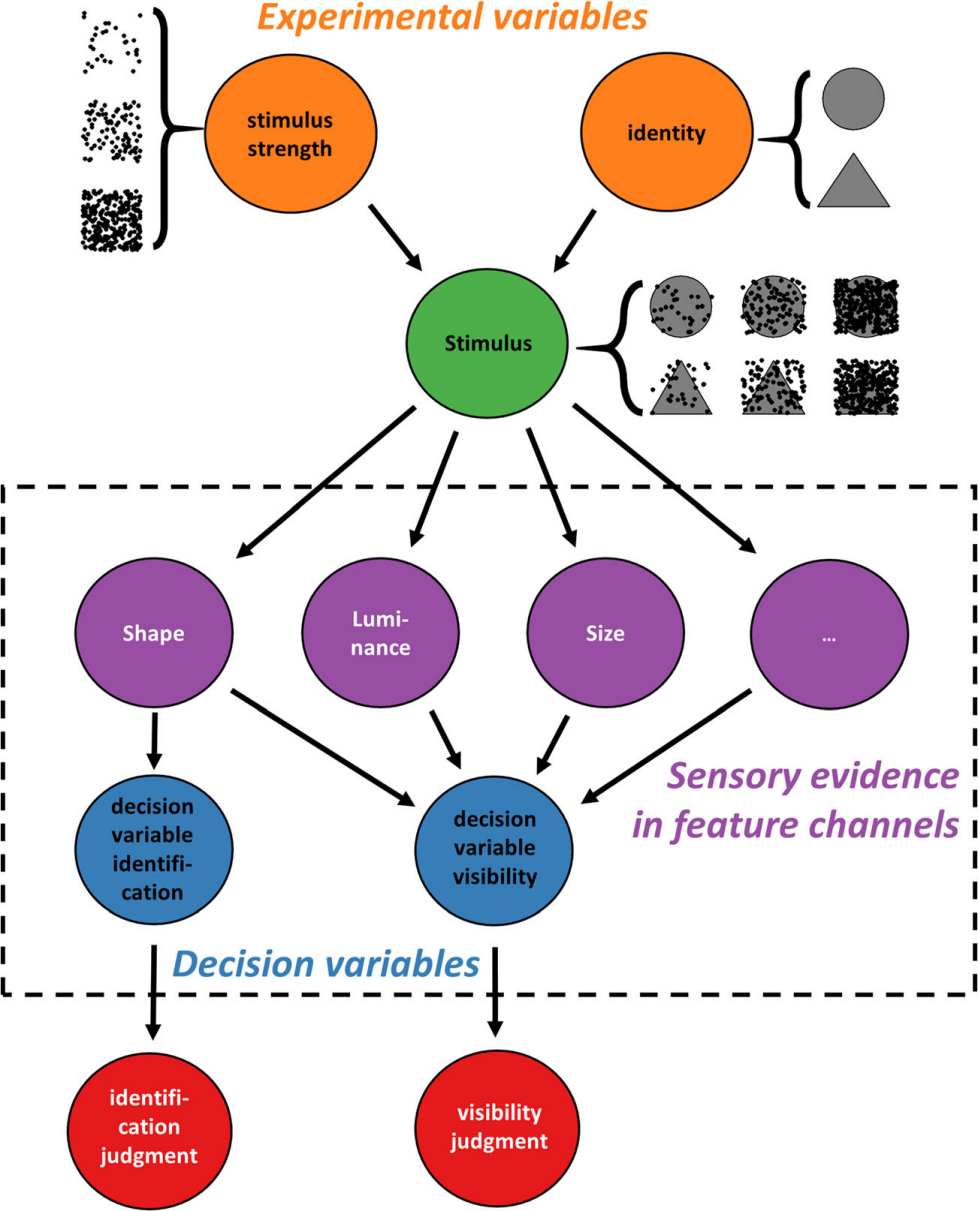
The weighted evidence and visibility model, adapted to describe visibility. The stimulus varies in two aspects: A feature relevant to the identification judgment (symbolized here as circle and a triangle and a manipulation of stimulus strength (symbolized by the noise dots). The stimulus creates sensory evidence about the shape of the stimulus, but also sensory evidence about the other features of the stimulus (e.g., its size or color), whose strength is informative about the strength of stimulation. The evidence about the identity of the stimulus is used to make an identification judgment. Visibility judgments are determined based on a combination of sensory evidence about the identity of the stimulus and the strength of evidence about identity-irrelevant features

The present study proposes that visibility judgments can be described by a novel interpretation of the weighted evidence and visibility model (WEV model; see Fig. 1). The WEV model was derived from signal detection theory (SDT; Green & Swets, 1966; Macmillan & Creelman, 2005; Wickens, 2002) and had been developed as a model of confidence in postmasked orientation judgments (Rausch et al., 2018; Rausch et al., 2020).

The main tenet is that subjective visibility is determined by a combination of sensory evidence relevant to the identification judgment as well as strength of evidence about identification-irrelevant features of the stimulus. In many psychophysical experiments, two characteristics of the stimulus are varied across trials: Observers need to select a response based on one varied characteristic of the stimulus, to which we refer as the identity of the stimulus. In addition, there is an experimental manipulation of stimulus strength (e.g., presentation duration or contrast), which is varied orthogonally to the identity of the stimulus. The visual system does not only represent the identity but also the other features to some degree (Marshall & Bays, 2013; Xu, 2010). This means that if observers are asked to report the shape of the stimulus, other features such as size, orientation, or color will be nevertheless represented within the brain. The strength of representation of the different features varies from trial to trial and may be to some degree independent of each other as the visual system processes visual features in parallel (Kyllingsbæk & Bundesen, 2007). The representation of the identity of the stimulus is used to select a response. However, the representations of identity-irrelevant features are not useless; they depend on the strength of stimulation, too and thus allow observers to estimate the reliability of their percept: When there is strong evidence about many features, observers will have a distinct experience of the stimulus, and report a high degree of visibility. When the evidence about many features of the stimulus is weak, observers will consider the visibility of the stimulus as low.

The WEV model expresses the hypothesis that visibility depends on sensory evidence relevant to the identification judgment and on the strength of evidence about identification-irrelevant features in formal terms. According to the WEV model, the stimulus is characterized by two experimental variables, the identity of the stimulus *S*_*id*_ ∈ {−1, 1} and the strength of the stimulus *S*_*s*_ ∈ {*S*_1_, *S*_2_, …, *S*_*n*_}. Participants select an identification response *R*_*id*_ ∈ {−1, 1} about the identity of the stimulus as well as a visibility judgment out of several possible ordered categories of visibility *R*_*v*_ ∈ {0, 1, 2, …, *v*_*max*_}.

For identification judgments, the WEV model assumes the same decision mechanism as SDT. The choice about the identity of the stimulus requires a comparison between the decision variable for the identification judgment *δ*_*id*_, usually referred to as sensory evidence, with the free criterion parameter *θ*_*id*_. The decision variable for the identification judgment *δ*_*id*_ is a random sample from a Gaussian distribution $\mathcal{N}$:

1 $$ {\delta}_{id}\sim \mathcal{N}\left(\mu =\frac{1}{2}\times {S}_{id}\times {S}_s,\sigma ={\sigma}_{id}\right) $$

*S*_*s*_ denotes the distance of the distributions generated by the two possible identities of the stimulus. If the standard deviation *σ*_*id*_ is fixed at 1, *S*_*s*_ is equivalent to the sensitivity parameter d’ of SDT. Participants are assumed to respond *R*_*id*_ =  − 1 if *δ*_*id*_ < *θ*_*id*_, and *R*_*id*_ = 1 otherwise.

Concerning visibility judgments, the choice selecting a specific degree of subjective visibility requires comparison of another decision variable *δ*_*v*_ against a set of criteria *θ*_*v*_. Each criterion delineates between two adjacent categories of visibility, e.g., participants select category 2 if *δ*_*v*_ falls between *θ*_*v*1_ (which separates category 1 and 2) and *θ*_*v*2_ (which separates category 2 and 3). For consistency with SDT, a separate set of criteria is assumed for each of the two response options. The decision variable for the visibility judgment *δ*_*v*_ is also a random sample from a Gaussian distribution:

2 $$ {\delta}_v\sim \mathcal{N}\left(\mu =\left(1-w\right)\times {\delta}_{id}+w\times {R}_{id}\times \left({S}_s-\overline{S_s}\right),\sigma ={\sigma}_v\right) $$

The parameter *w* captures the degree to which participants rely on sensory evidence about the identity or on identity-irrelevant sensory evidence for subjective visibility. If *w* = 0, *δ*_*v*_ depends only on the decision variable for the identification judgment *δ*_*id*_. If *w* = 1, *δ*_*v*_ depends only on the strength of stimulation *S*_*s*_, but not on *δ*_*id*_*.* The term ${R}_{id}\times \left({S}_s-\overline{S_s}\right)$ ensures that strong stimuli relative to the other stimuli in the experiment tend to shift the location of the distribution in a way that high visibility is more likely, and likewise, weak stimuli tend to shift the location of the distribution in a way that the probability of low visibility increases. ${\overline{S}}_s$ denotes the mean of *S*_*s*_ across all conditions of the experiment. The standard deviation *σ*_*v*_ quantifies the amount of unsystematic variability contributing to visibility judgments but not to identification judgments. The unsystematic variability may stem from different sources, including the uncertainty in the estimate of stimulus strength and criterion setting as well as noise inherent to metacognitive processes.

The WEV model is distinct from two other extensions of SDT: multi-dimensional SDT (King & Dehaene, 2014) and the response-congruent evidence model (Maniscalco et al., 2016; Peters et al., 2017). The WEV model and multi-dimensional SDT share the idea that the representations of multiple features determine visibility judgments. Yet, the WEV model assumes many different categories of subjective visibility, not only a binary decision if the stimulus is present or absent. The response-congruent evidence model is a recent extension of multi-dimensional SDT for confidence judgments. The response-congruent evidence model assumes a separate dimension of evidence for each of the two choice options. It asserts that while the identification judgment is based on both dimensions of evidence, confidence is sensitive only to evidence in favor of the selected choice, and neglects evidence against the choice (Zylberberg et al., 2012). In contrast, according to the WEV model, evidence in favor of the choice and against the choice both inform the identification decision, and subjective visibility and confidence are influenced by sensory evidence about stimulus features unrelated to the choice.

The WEV model is also reminiscent but not identical to the partial awareness hypothesis (Kouider et al., 2010). According to the partial awareness hypothesis, conscious access to each feature of the stimulus is assumed to be all-or-nothing. Partial awareness of a stimulus is a state when some features of that stimulus are consciously accessible while other features cannot be accessed. A state of partial awareness was proposed as an explanation why participants occasionally report a medium degree of visibility, although global workspace theory postulates that conscious access is all-or-nothing (Sergent & Dehaene, 2004). In contrast to the partial awareness hypothesis, the WEV model conceives all decision variables to be continuous, which accounts naturally for intermediate degrees of visibility.

### Modelling the distinction between subjective visibility and confidence

If the WEV model is a suitable model not only of confidence but also of subjective visibility, fitting the WEV model independently to confidence and visibility judgements can be used to investigate the mechanism of why visibility is sometimes distinct from confidence. The reason is that three previously proposed hypotheses can all be mapped to different parameters of the WEV model. We refer to these hypotheses as (a) the feature hypothesis (Rausch et al., 2015; Rausch & Zehetleitner, 2016), (b) the metacognitive hypothesis (Charles et al., 2013; Jachs et al., 2015; Overgaard & Sandberg, 2012), and (c) the criterion hypothesis (Wierzchoń et al., 2012).

According to the feature hypothesis, subjective visibility may depend more strongly on sensory evidence about identity-irrelevant features than confidence does. The parameter *w* describes the relative weight of the sensory evidence about identity-irrelevant features. Thus, the feature hypothesis predicts that the relative weight the observer places on sensory evidence about identity-irrelevant features is greater for subjective visibility and less for confidence. For example, when observers rate the visibility of the stimulus, they may retrospectively attend all features of the stimulus stored in visual working memory, while for confidence, they might select only the identity-relevant features.

Concerning the metacognitive hypothesis, two distinct metacognitive processes involved in confidence but not in visibility have been suggested: First, while visibility judgments may be directly informed by visual experience, confidence may depend on a more error-prone metacognitive process that relates visual experience to task performance (Overgaard & Sandberg, 2012). The parameter *σ*_*v*_ quantifies the amount of unsystematic noise contributing to subjective reports. but not to identification judgments. Thus, if the processes underlying confidence are in fact more error-prone than those underlying visibility, when the WEV model is fitted independently to confidence judgments and visibility judgments, *σ*_*v*_ estimated from confidence should be larger than *σ*_*v*_ estimated from visibility. Second, confidence may depend on a metacognitive system that operates independently from visual experience (Charles et al., 2013; Charles et al., 2014; Jachs et al., 2015). As *σ*_*v*_ is sensitive to the amount of noise generated by metacognitive processes, if visibility and confidence rely in parts on separate metacognitive processes, and if these two metacognitive systems are not completely on par with each other in terms of noise, visibility and confidence are again expected be associated with different *σ*_*v*_ parameters.

The criterion hypothesis asserts that the difference between visibility and confidence can entirely be explained by participants applying different criteria to the same decision variable, but visibility judgments impose a more conservative reporting strategy than confidence judgments (Wierzchoń et al., 2012). Accordingly, it would be expected that fitting the WEV model to visibility and confidence results in different sets of *θ*_*v*_.

### Rationale of the present study

The present study investigated whether the WEV model provides a suitable account of visibility judgments. As the only reliable way of identifying computational models of confidence is by quantitative model comparisons (Adler & Ma, 2018), model fits of the WEV model were compared with a series of models of confidence that, for the purpose of the present study, were treated as models of subjective visibility. In addition, we investigated whether visibility judgments and decisional confidence are associated with the same sets of parameters of the WEV model.

To accomplish these aims, we asked observers in Experiment 1 to perform a postmasked orientation discrimination task, the same task for which the WEV was originally developed as a model of confidence (Rausch et al., 2018). After each orientation judgment, observers reported the subjective visibility of the stimulus on a visual analogue scale (Rausch & Zehetleitner, 2014; Sergent & Dehaene, 2004). In Experiment 2, observers again performed the postmasked orientation task as in Experiment 1, but this time they reported both their subjective visibility and their degree of confidence in the orientation discrimination task after each single trial (Zehetleitner & Rausch, 2013). This procedure allowed us to contrast model fits to visibility and confidence judgments based on identical perceptual and attentional processing of the target. In both experiments, the stimulus-onset asynchrony (SOA) between target stimulus and postmask was varied to manipulate stimulus strength.

In both experiments, the WEV model was compared against a series of models of confidence in binary perceptual choices: the rating model of signal detection theory (Green & Swets, 1966; Macmillan & Creelman, 2005; Wickens, 2002), the noisy SDT model (Maniscalco & Lau, 2016), the postdecisional accumulation model (Pleskac & Busemeyer, 2010), the two-channel model (Rausch & Zehetleitner, 2017), the response-congruent evidence model (Maniscalco et al., 2016), the constant noise and decay model (Maniscalco & Lau, 2016), and the two-dimensional Bayesian model (Aitchison et al., 2015). In addition, we examined whether model fitting came to the same conclusions when the variance of the decision variable was constant, when the variance increased with SOA, and when the variance was different between the two possible identities of the target stimulus. Models were fitted to the combined distributions of identification judgments and visibility or confidence—thus, differences in objective task performance were accounted for by explicitly modelling objective performance in addition to visibility. As the main aim of the present study was to account for the regularities underlying visibility, we do not consider sequential sampling models (Ratcliff et al., 2016), which were designed to explain the dynamics of the decision process. Yet reaction times were analyzed to investigate whether it is legitimate to exclude reaction times from modelling (see Supplementary Figs. S1 and S2).

It was hypothesized that if the WEV model provides the best account of subjective visibility, the WEV model should be associated with better goodness-of-fit indices than any of the competing models. In addition, with respect to Experiment 2, we tested three hypotheses about the mechanism underlying the distinction between subjective visibility and confidence. According to the feature hypothesis, the weight parameter would be expected to be greater for visibility than for confidence. If there were metacognitive processes involved exclusively in confidence but not in visibility, as proposed by the metacognitive hypothesis, we would expect different noise parameters between confidence and visibility. If the criterion hypothesis was correct, the model fits should reveal systematically different sets of criteria for visibility and for confidence.

## Experiment 1

### Methods

#### Participants

Thirty-four participants (four males, 30 female) were recruited using the ORSEE Online Recruitment System (Greiner, 2015). Their age ranged between 18 and 44 years (*M* = 21.7). All participants reported normal or corrected-to-normal vision, no history of neuropsychological or psychiatric disorders and not to be on psychoactive medication. All participants gave written informed consent and received either course credits or €8 per hour as compensation for participation.

#### Apparatus and stimuli

The experiment was performed in a darkened room. The stimuli were presented on a Display++ LCD monitor (Cambridge Research Systems, UK) with a screen diagonal of 81.3 cm, set at a resolution of 1,920 × 1,080 pixels and a refresh rate of 120 Hz. The distance between the monitor and the participant was approximately 60 cm. The experiment was conducted using PsychoPy v.1.83.04 (Peirce, 2007, 2009) on a Fujitsu ESPRIMO P756/E90+ desktop computer with Windows 8.1. The target stimulus was a square (size 3° × 3°), textured with a sinusoidal grating with one cycle per degree of visual angle (maximal luminance: 64 cd/m^2^; minimal luminance: 21 cd/m^2^). The postmask consisted of a square (4° × 4°) with a black-and-white checkered pattern (0 cd/m^2^ and 88 cd/m^2^) consisting of five columns and rows. All stimuli were presented at fixation against a gray (44 cd/m^2^) background. The orientation of the grating varied randomly between horizontal or vertical. Participants reported the orientation of the target by pressing “A” on the keyboard when the target was horizontal and “S” when the target was vertical. The subjective visibility was reported by moving an index on a continuous scale using a Cyborg V1 joystick (Cyborg Gaming, UK). A continuous scale was used to record the maximum amount of information per single measurement (Rausch & Zehetleitner, 2014).

#### Experimental procedure

**Fig. 2:**
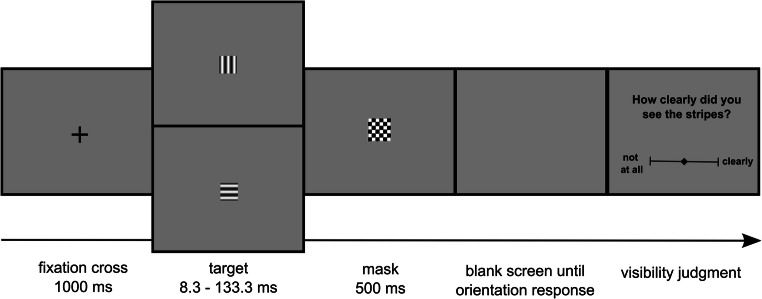
Trial structure of Experiment 1

Figure 2 depicts the time course of one trial. Each trial began with the presentation of a fixation cross for 1 s. Then the target stimulus was shown for 8.3, 16.7, 33.3, 66.7, or 133.3 ms until it was replaced by the postmask. The postmask was presented for maximally 500 ms. When participants did not respond to the orientation task within 500 ms, the postmask disappeared, and an empty screen was shown until participants responded to the orientation task. After that, the question “How clearly did you see the stripes?” was displayed on screen. Participants reported the visibility of the stimulus using a visual analogue scale and a joystick, meaning that participants selected a position along a continuous line between two end points by moving a cursor. The end points were labelled as “not at all” and “clearly.” Participants confirmed a position on the continuous line by pulling the trigger of the joystick with their index finger. Finally, if the orientation response was wrong, the trial ended by the presentation of the word “error” for 1 s.

#### Design and procedure

Participants were instructed to report the orientation of the grating and the visibility of the stimulus as accurately as possible without time pressure. The experiment consisted of one training block and 11 experimental blocks of 50 trials each. Each SOA featured 10 times in each block in random order. The orientation of the target stimulus varied randomly across trials.

#### Model specification

Eight different models of confidence were fitted to the combined distributions of orientation identification judgments and discretized subjective visibility separately for each single participant:

(i) the SDT rating model
(ii) the noisy SDT model
(iii) the WEV model
(iv) the two-channel model
(v) the postdecisional accumulation model
(vi) the constant noise and decay model
(vii) the response-congruent evidence model
(viii) the 2D Bayesian model

Table 1 provides an overview of the parameters of the eight models. Models (i)–(v) were preregistered online before data collection; models (vi)–(viii) were added post hoc to allow for a more comprehensive model comparison.

**Table 1:** Free parameters of each model of confidence

Model	Parameter	Interpretation
All models	*θ*_*v01*_*, θ*_*v02*_*, θ*_*v03*_*, θ*_*v04*_*, θ*_*v11*_*, θ*_*v12*_*, θ*_*v13*_*, θ*_*v14*_	Criteria separating between two adjacent rating responses. One set for each of the two possible identity of the stimulus.
All models except for the 2D Bayesian model	*S*_*S1*_*, S*_*S2*_*, S*_*S3*_*, S*_*S4*_*, S*_*S5*_	Identification sensitivity for each SOA
	*θ*_*id*_	Criterion for the identification response
Noisy SDT model	*σ*_*v*_	Noise superimposed on rating response
WEV model	*w*	Degree to which ratings rely on sensory evidence about the identity or on strength of evidence about identification-irrelevant features of the stimulus
	*σ*_*v*_	Noise superimposed on rating responses
Two-channel model	*a*	Fraction of signal available to the channel informing rating response, relative to the signal available to the first channel
Postdecisional accumulation model	*b*	Amount of postdecisional accumulation relative to the evidence available at the time of the identification choice
Constant noise and decay model	*ρ*_*1*_*, ρ*_*2*_*, ρ*_*3*_*, ρ*_*4*_*, ρ*_*5*_	Signal reduction parameter for each SOA
	*σ*_*v*_	Noise superimposed on rating responses
2D Bayesian model	*s*	Perceptual noise parameter
	*λ*	Lapse rate
Variants of models where the variance of the decision variable increased with SOA	*k*	Increase of the variance of *δ*_*id*_with stimulus strength
Variants of models where the variance of the decision variable depended on the identity of the stimulus	*r*_*id*_	Dissimilarity of variabilities of the decision variable associated with the two possible identities of the stimulus

The SDT rating model, the noisy SDT model, the two-channel model, the postdecisional accumulation model, and the constant noise and decay model all assume the same mechanism for the identification decision as we have described above for the WEV model. The models are different only in the way how *δ*_*v*_ is calculated. Concerning the stimulus strength *S*_*s*_, a separate free parameter was fitted for each SOA. Concerning the standard deviation *σ*_*id*_, model fitting was repeated with three different assumptions about the variability of *δ*_*id*_ to ensure that the results were robust across different assumptions about noise. For the first set of analyses, the standard deviation of *σ*_*id*_ was fixed at 1 for both identities of the stimulus and for all SOAs. For the second set analyses, the variability of *δ*_*id*_ could vary depending on *S*_*id*_: An additional parameter r_id_ characterized the relationship between the variability of *δ*_*id*_ associated with the two possible identities of the stimulus:

3 $$ {\sigma}_{id}={r_{id}}^{S_{id}}. $$

Finally, a third run of analyses examined whether the same results were obtained when ${\sigma}_{id}^2$ increased with the square of *S*_*s*_:

4 $$ {\sigma}_{id}=\sqrt{1+k\times {S_s}^2}. $$

*k* is a free parameter quantifying the slope of the increase of variance with *S*_*s*_^2^.

##### SDT rating model

According to SDT, the decision variables for identification and visibility are identical:

5 $$ {\delta}_v={\delta}_{id}. $$

##### Noisy SDT model

Conceptually, the noisy SDT model reflects the idea that confidence/visibility is informed by the same sensory evidence as the identification choice, but confidence/visibility is affected by additive, nonperceptual noise. Therefore, *δ*_*v*_ is also sampled from a Gaussian distribution, with a mean equal to the decision variable *δ*_*id*_ and the standard deviation *σ*_*v*_, which is an additional free parameter:

6 $$ {\delta}_v\sim \mathcal{N}\left(\mu ={\delta}_{id},\sigma ={\sigma}_v\right). $$

##### Two-channel model

Conceptually, the two-channel model represents the case where confidence is informed by a second sample of sensory evidence, independent from the identification decision (e.g., Pasquali et al., 2010). Therefore, *δ*_*v*_ is again sampled from a Gaussian distribution, but now independently from *δ*_*id*_:

7 $$ {\delta}_v\sim \mathcal{N}\left(\upmu =\frac{1}{2}\times {S}_{id}\times {S}_s\times a,\sigma =1\right). $$

The free parameter *a* expresses the fraction of signal available to the second channel relative to the signal available to the first channel.

##### Postdecisional accumulation model

This model was inspired by two-stage signal detection theory, according to which observers continue to accumulate evidence after the decision for a fixed time interval (Pleskac & Busemeyer, 2010). To ensure comparability with the other models considered here, we used a model that represents the conceptual idea of ongoing accumulation of evidence, but is not fitted to reaction time data. According to the model, *δ*_*v*_ is again sampled from a Gaussian distribution:

8 $$ {\delta}_v\sim \mathcal{N}\left(\upmu ={\delta}_{id}+{S}_{id}\times {S}_s\times b,\sigma =\sqrt{\mathrm{b}}\right). $$

The free parameter *b* indicates the amount of postdecisional accumulation relative to the amount of evidence available at the time of the identification decision. The term *S*_*id*_ ensures that postdecisional accumulation tends to decrease *δ*_*v*_ when *S*_*id*_ =  − 1, and to increase *δ*_*v*_ when *S*_*id*_ = 1. The standard deviation equals $\sqrt{\mathrm{b}}$ because both the mean and the variance of the decision variable increase linearly with time in drift diffusion processes (Pleskac & Busemeyer, 2010).

##### Constant noise and decay model

Conceptually, the constant noise and decay model reflects the idea that confidence/visibility is informed by the same evidence as the identification choice, but confidence or visibility judgments are also distorted by a multiplicative decay of sensory evidence, which is specific to each SOA, and additional late noise as in noisy SDT. According to the model, *δ*_*v*_ is also sampled from a Gaussian distribution with the standard deviation *σ*_*v*_. The mean of *δ*_*v*_ depends on *δ*_*id*_, but *δ*_*id*_ is reduced by multiplication with a signal reduction parameter *ρ*_*S*_. The signal reduction parameter *ρ*_*S*_ is a free parameter specific to each SOA and is bounded between 0 and 1:

9 $$ {\delta}_v\sim \mathcal{N}\left(\mu ={\delta}_{id}\times {\rho}_S,\sigma ={\sigma}_v\right). $$

##### Response-congruent evidence model

The response-congruent evidence model assumes a different decision mechanism for the identification judgment than the WEV model: The response-congruent evidence model assumes two separate decision variables for the identification judgment, each belonging to one possible identity of the stimulus:

10 $$ {\displaystyle \begin{array}{c}{\delta}_{id-}\sim \mathcal{N}\left(\mu =\frac{1}{2}\times \left(1-{S}_{id}\right)\times {S}_s-{\theta}_{id},\sigma ={\sigma}_{id}\right)\\ {}{\delta}_{id+}\sim \mathcal{N}\left(\mu =\frac{1}{2}\times \left(1+{S}_{id}\right)\times {S}_s+{\theta}_{id},\sigma ={\sigma}_{id}\right)\end{array}} $$

The parameter θ_id_ reflects the a priori bias in favor of *R*_*id*_ = 1. Participants are assumed to respond *R*_*id*_ =  − 1, when *δ*_*id*−_ > *δ*_*id*+_, and *R*_*id*_ = 1 if *δ*_*id*−_ < *δ*_*id*+_. Visibility judgments are only based on the decision variable pertaining to the selected response: When *R*_*id*_ =  − 1, *δ*_*id*−_ is compared against a series of visibility criteria *θ*_*v*−_ to select a specific degree of confidence; and when *R*_*id*_ = 1, the comparison is based on *δ*_*id*+_ as well as a second set of criteria *θ*_*v*+_. The parameter θ_id_ was not present in the original version of the model (Peters et al., 2017), but we included it the present study because θ_id_ strongly improved model fit and allows for a more direct comparison between the response-congruent evidence model and the models derived from signal detection theory, which all include an equivalent bias parameter.

##### 2D Bayesian model

According to the 2D Bayesian model, there are also two separate decision variables, *δ*_*id*−_ and *δ*_*id*+_, referred to as “sensory signals” by Aitchison et al. (2015). These two separate decision variables may be interpreted as the output of two independent feature channels, each tuned to one out of the possible identities of the stimulus. While there is some evidence for stochastically independent feature channels when stimuli are presented for brief time periods (Kyllingsbæk & Bundesen, 2007), uncorrelated feature channels might be an oversimplification (Klein, 1985).

11 $$ {\displaystyle \begin{array}{c}{\delta}_{id-}\sim \mathcal{N}\left(\mu =\frac{1}{2}\times \left(1-{S}_{id}\right)\times \Delta t,\sigma =s\right)\\ {}{\delta}_{id+}\sim \mathcal{N}\left(\mu =\frac{1}{2}\times \left(1+{S}_{id}\right)\times \Delta t,\sigma =s\right)\end{array}} $$

Δ*t* denotes the physical stimulus-onset asynchrony and *s* is a free noise parameter. The model assumed that the observer’s choices about the identity of the stimulus and about the visibility depend on the posterior probability of the identity of the stimulus given the decision variables *P*(*S*_*id*_| *δ*_*id*−_, *δ*_*id*+_):

12 $$ P\left({S}_{id}=1|{\updelta}_{\mathrm{id}-},{\updelta}_{\mathrm{id}+}\right)=\frac{\sum_tP\left({\updelta}_{\mathrm{id}-}|\Delta \mathrm{t}=\mathrm{t},s,{S}_{id}=1\right)P\left({\updelta}_{\mathrm{id}+}|\Delta \mathrm{t}=\mathrm{t},s,{S}_{id}=1\right)}{\sum \limits_{t,i}P\left({\updelta}_{\mathrm{id}-}|\Delta \mathrm{t}=\mathrm{t},s,{S}_{id}=i\right)P\left({\updelta}_{\mathrm{id}+}|\Delta \mathrm{t}=\mathrm{t},s,{S}_{id}=i\right)}. $$

The formula assumes that observers apply a flat prior across the discrete set of SOAs as well as across the two possible identities of the stimulus. In many classic detection and discrimination tasks, signal detection theory models are equivalent to Bayesian models (Ma, 2012). However, the 2D Bayesian model as defined here is not equivalent to the standard SDT model as described above because the 2D Bayesian model does not rely simply on the difference between δ_id−_ and δ_id+_; instead, the formula requires the sum of the probability of δ_id−_ and δ_id+_ given SOA *P*(δ_id − ,_δ_id+_| Δt = t ) over SOAs. A specific identity and degree of visibility are chosen by comparing the posterior probability *P*(*S*_*id*_ = 1| *δ*_*id*−_, *δ*_*id*+_) against a set of criteria *θ*. The 2D Bayesian model assumes that the possible identities and degrees of visibility form an ordered set of decision options. Each criterion delineates two adjacent decision options—for example, participants choose to respond that the identity is 1 and visibility is 1 if *P*(*S*_*id*_ = 1| δ_id−_, δ_id+_) is smaller than the criterion associated with Identity 1 and Visibility 2, and at the same time *P*(*S*_*id*_ = 1| δ_id−_, δ_id+_) is greater than the criterion for Identity 0 and Visibility 1. Finally, it is assumed that observers do not always gave the same response as they intend to. When a lapse occurs, identification and visibility responses are assumed to be random with equal probabilities. The lapse rate *λ* is an additional free parameter.

#### Model fitting

The fitting procedure involved the following computational steps: First, the continuous visibility ratings were discretized by dividing the continuous scale into five partitions of equal length. Five categories of visibility meant that there were 11 free parameters for the 2D Bayesian model; 14 for the SDT model and the response-congruent evidence model; 15 for the noisy SDT model, two-channel model, and the postdecisional accumulation model; 16 free parameters for the WEV model; and 20 for the constant noise and decay model.

Then, the frequency of each visibility category was calculated for each orientation of the stimulus and each orientation response. For each model, the set of parameters was determined that minimized the negative log-likelihood of the data given the model (see Supplementary Table S1). For this purpose, we used a coarse grid search to identify five promising sets of starting values for the optimization procedure. Then, minimization of the negative log-likelihood was performed using a general SIMPLEX minimization routine (Nelder & Mead, 1965) for each set of starting values. To avoid local minima, the optimization procedure was restarted four times.

#### Statistical analysis

To assess the relative quality of the eight candidate models, we calculated the Bayes information criterion (Schwarz, 1978) and the AICc (Burnham & Anderson, 2002), a variant of the Akaike information criterion (Akaike, 1974) using the negative likelihood of each model fit with respect to each single participant and the trial number. For statistical testing, we used Bayes factors as implemented in the R package *BayesFactor* (Morey & Rouder, 2015), a Bayesian equivalent of paired *t* tests (Dienes, 2011; Rouder et al., 2009). A Cauchy distribution with a scale parameter of 1 was assumed as prior distribution for the standardized effect size δ, which is given by the mean difference divided by the standard deviation of the difference. The Cauchy prior over standardized effect sizes had been recommended as default in psychology (Rouder et al., 2009). The strength of statistical evidence was interpreted according to an established guideline (Burnham & Anderson, 2002; Lee & Wagenmakers, 2013). In addition, 95% HDI intervals were created using10^6^ samples from the posterior. All analyses were conducted using the free software R (R Core Team, 2014).

### Results

**Fig. 3:**
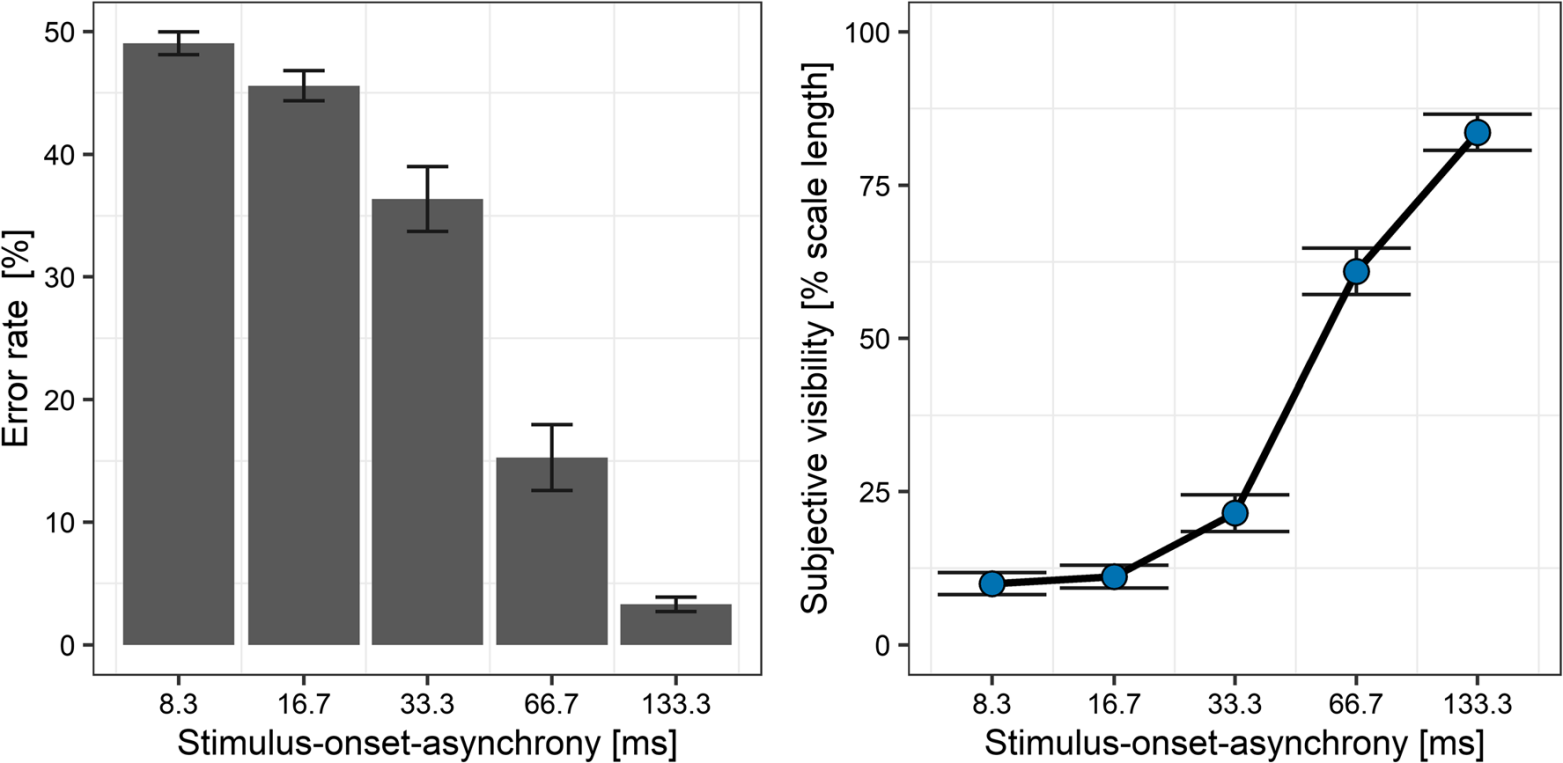
Error rate in the orientation task (left panel) and subjective visibility (right panel) as a function of stimulus-onset asynchrony (*x*-axis) in Experiment 1. Bars and symbols indicate observed means. Error bars indicate 1 *SEM*

Three participants were excluded from the analysis because they did not perform the identification task above chance level, all BF_10_s ≤ .24. For the remaining 31 participants, the error rate ranged between chance level at an SOA of 8.3 ms (*M* = 49.0%, *SD* = 5.2) and ceiling at the maximum SOA of 133.3 ms (*M* = 3.3%, *SD* = 3.3, see Fig. 3, left panel). Subjective visibility averaged 10.0% (*SD* = 10.1) of the width of the visual analogue scale at the SOA of 8.3 ms and increased to a mean of 83.6% (*SD* = 16.2) at an SOA of 133.3 ms (see Fig. 3, right panel). Reaction times as a function of visibility is depicted in Supplementary Fig. S1, showing a strong overlap of reaction time distributions across degrees of visibility. In addition, while visibility was very strongly associated with SOA, reaction times were also quite similar across SOAs (see Supplementary Fig. S2).

#### Model fits

**Fig. 4:**
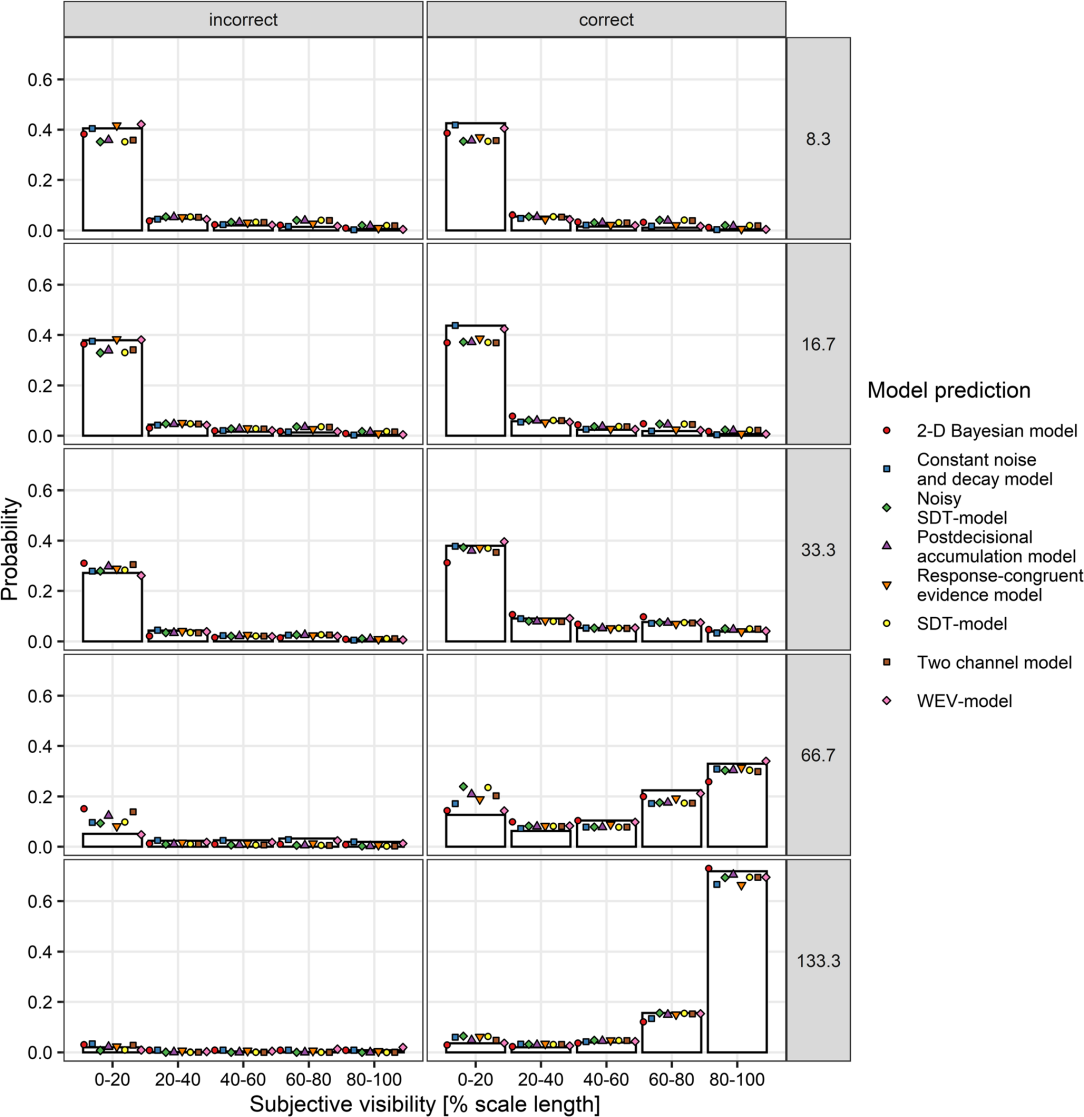
Distribution of subjective visibility depending on stimulus-onset-asynchrony (rows) and accuracy of the identification judgments (columns) in Experiment 1. Symbols show the prediction of the different models based on the sets of parameters identified during model fitting assuming constant variances of the decision variable

Figure 4 depicts the observed distribution of subjective visibility for correct and incorrect responses and for each stimulus-onset asynchrony and as well as the predicted distributions for each of the eight models. It shows that the WEV model and the constant noise and decay model provided the best fit to the probability of low visibility at lower SOAs.

**Fig. 5:**
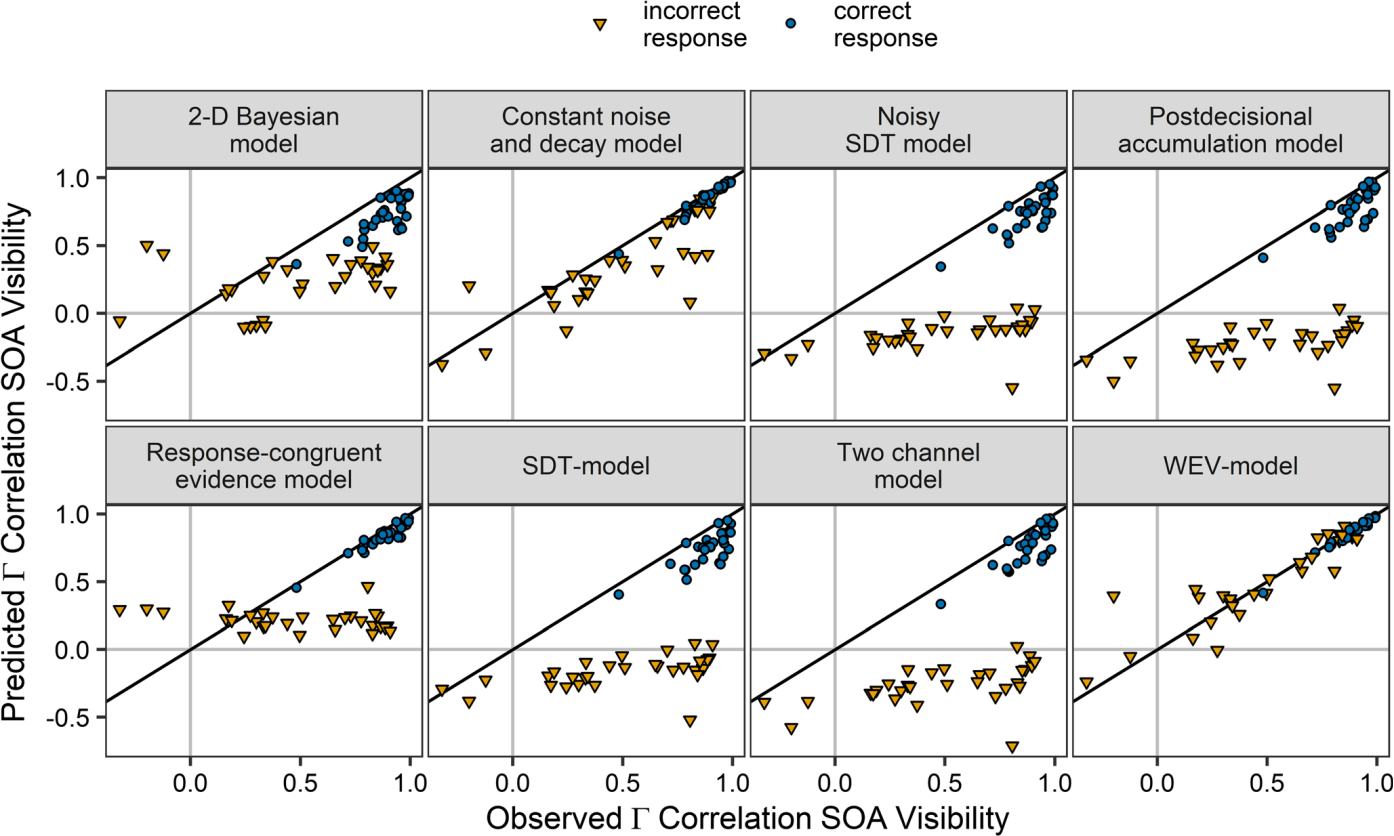
Gamma correlation coefficients between SOA and visibility derived from the model fits for each of the eight models of confidence in separate panels as a function of the observed gamma correlation coefficients for correct trials (circles) and incorrect trials (triangles). Each symbol represents the data from one participant

Figure 5 shows that the WEV model was able to account for the pattern of correlations between SOA and visibility. In contrast, the constant noise and decay model tended to underestimate the correlation in incorrect trials. The other models also did not account for the variability of the correlation in incorrect trials across participants.

#### Formal modal comparisons

**Fig. 6:**
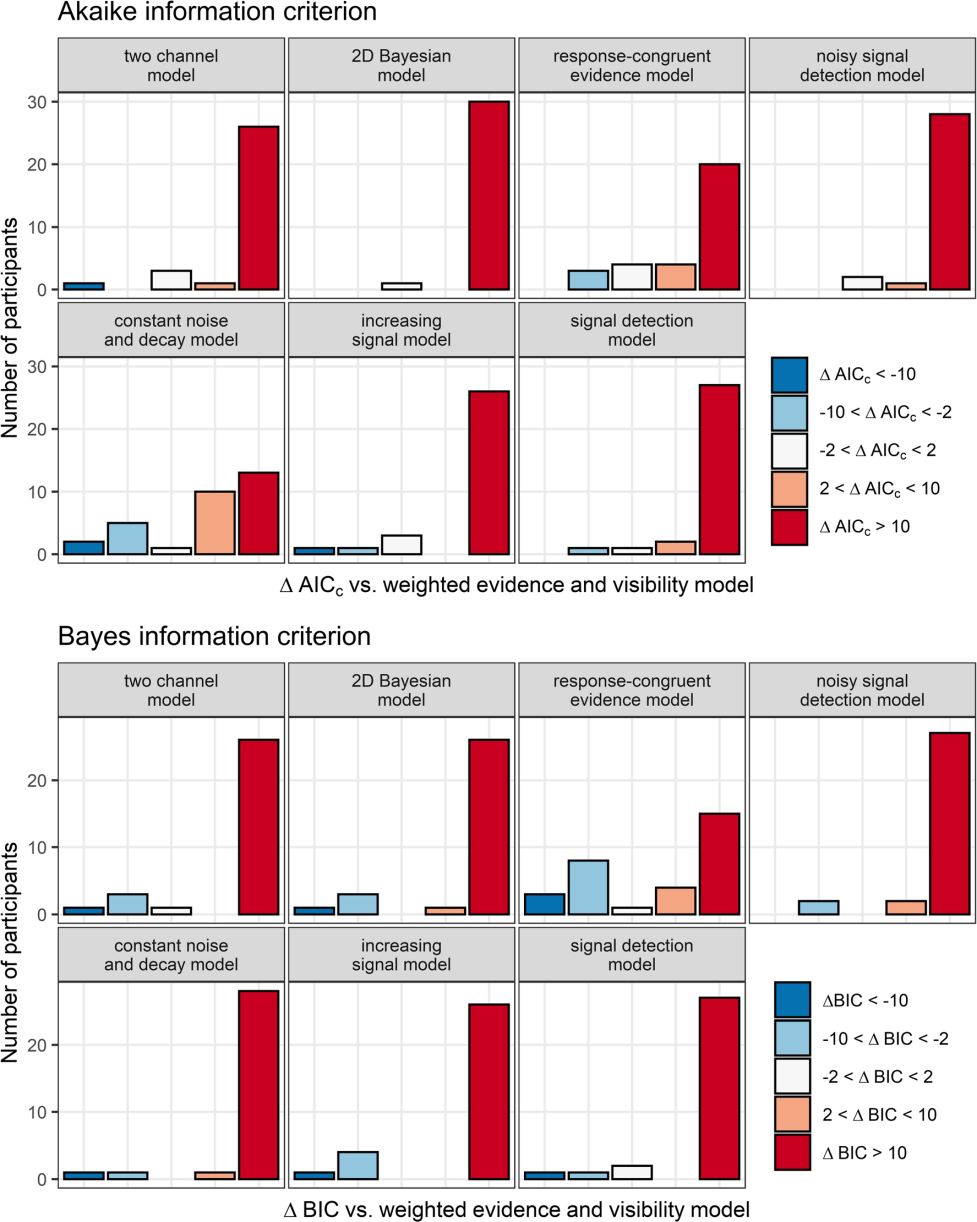
Model fits to subjective visibility. The different panels depict the frequency of AIC_c_- and BIC differences when the WEV model was compared with each of the seven other models, assuming constant variances of the decision variable. AIC_c_ and BIC differences were assorted into categories based on an established guideline for interpretation (Burnham & Anderson, 2002)

Formal model comparisons revealed that the best fits to the data were obtained by the WEV model both in terms of AIC_c_, and BIC independently of whether the variances of the decision variables were assumed to be constant (see Fig. 6), to be different between the two possible identities of the stimulus, or to increase with SOA.

For constant variances, Bayes factors indicated that the evidence that the WEV model performed better than the constant noise and decay model was strong in terms of AIC_c_, *M*_*ΔAIC*_ = 13.4, 95% HDI [4.8, 20.8], BF_10_ = 14.1, and extremely strong in terms of BIC, *M*_*ΔBIC*_ = 30.1, 95% HDI [21.1, 37.6], BF_10_ = 6.9×10^5^. Likewise, the evidence that the WEV model performed better than the response-congruent evidence model was extreme in terms of AIC_c_, *M*_*ΔAIC*_ = 28.1, 95% HDI [15.1, 39.1], BF_10_ = 472.2, and very strong in terms of BIC, *M*_*ΔBIC*_ = 19.7, 95% HDI [7.0, 30.7], BF_10_ = 13.3. There was also extremely strong evidence that the WEV model performed better than each of the other models in terms of AIC_c_ and BIC, all *M*_*ΔAIC*_s ≥ 87.5, *M*_*ΔBIC*_s ≥ 66.4, BF_10_s ≥ 4.0×10^4^. Descriptive statistics of the fitted parameters of the WEV model are found in Supplementary Table S2.

When different variances associated with the two stimulus identities were assumed, there was again very strong evidence that the WEV model performed better than the constant noise and decay model in terms of AIC_c_, *M*_*ΔAIC*_ = 15.4, 95% HDI [6.8, 22.8], BF_10_ = 50.4, and extremely strong evidence in terms of BIC, *M*_*ΔBIC*_ = 32.1, 95% HDI [23.2, 39.6], BF_10_ = 2.5×10^6^. There was also extremely strong evidence that the WEV model performed better than the response-congruent evidence model given different variances between stimulus identities, AIC_c_: *M*_*ΔAIC*_ = 35.7, 95% HDI [21.2, 47.8], BF_10_ = 3.0×10^3^, BIC: *M*_*ΔBIC*_ = 27.3, 95% HDI [13.0, 39.4], BF_10_ = 105.8. There was moderate evidence that the WEV model with different variances for different stimulus identities provided a better fit to the data than the version of the WEV model with constant variances in terms of AIC_c_: *M*_*ΔAIC*_ = 8.8, 95% HDI [3.9, 13.0], BF_10_ = 5.0, but the evidence was not conclusive in terms of BIC, *M*_*ΔBIC*_ = 4.6, 95% HDI [−0.1, 8.9], BF_10_ = 0.90.

When the variances were assumed to increase as a function of SOA, Bayes factors once again indicated strong evidence that the WEV model performed better than the constant noise and decay model in terms of AIC_c_, *M*_*ΔAIC*_ = 12.0, 95% HDI [4.6, 18.4], BF_10_ = 20.1, and extremely strong evidence in terms of BIC, *M*_*ΔBIC*_ = 28.7, 95% HDI [21.0, 35.1], BF_10_ = 5.3×10^6^. Concerning the response-congruent evidence model, the evidence that the WEV model performed better was extremely strong in terms of AIC_c_, *M*_*ΔAIC*_ = 25.5, 95% HDI [12.5, 36.5], BF_10_ = 140.9, and moderate in terms of BIC, *M*_*ΔBIC*_ = 17.1, 95% HDI [4.5, 28.2], BF_10_ = 4.8. While the evidence was not conclusive whether the WEV model with SOA-dependent variances provided a better account for the data than the version of the WEV model with constant variances in terms of AIC_c_: *M*_*ΔAIC*_ = 3.9, 95% HDI [−1.6, 8.9], BF_10_ = 0.37, there was moderate evidence against a difference in terms of BIC, *M*_*ΔBIC*_ = 4.6, 95% HDI [−0.1, 8.9], BF_10_ = 0.14.

#### Model recovery analysis

To investigate whether one of the other models could have been misclassified as WEV model, 500 simulations were performed based on the second-best and the third-best performing model (i.e., the response-congruent evidence model and the constant noise and decay model assuming, again, constant variances of the decision variables). First, we sampled with replacements from the participants of Experiment 1 the same number of participants. Then, for each simulated subject, the parameter sets obtained during model fitting were used to create the same number of trials as in the real experiment. Both the generative model and the WEV model were fitted to the data of each simulated participant, after which Bayes factors were again used to test whether the simulated data were classified correctly as evidence in favor of the generative model, or incorrectly in favor of the WEV model. Supplementary Fig. S3 shows that not a single simulated data set based on the response-congruent evidence model was classified as providing evidence for the WEV model, and only one data set based on the constant noise and decay model resulted in false evidence for the WEV model in terms of BIC, but again not a single one did in terms of AIC_c_. Supplementary Fig. S3 also shows that compelling evidence was rare for BIC when the data was generated according to the constant noise and decay model; however, if the AIC_c_ was used or if the generative model was the response-congruent evidence model, the vast majority of simulations resulted in compelling evidence for the correct model (see also Supplementary Fig. S4).

### Discussion

The present experiment suggests that the WEV model provides a better account of visibility judgments in a postmasked orientation task than the SDT rating model, the noisy SDT model, the postdecisional accumulation model, the two-channel model, the two-dimensional Bayesian model, the response-congruent evidence model, and the constant noise and decay model. These models seemed to be specifically unable to account for the correlation between SOA and visibility: Non-WEV models tended to underestimate the correlation between SOA and visibility in incorrect trials, and all models except for the constant noise and decay model were not able to reproduce the interindividual variability of the correlations. At least for experiments with a strong correlation between stimulus strength and visibility or a strong variability of the correlation between stimulus strength and visibility, the WEV model seems to be the best option to model visibility judgments.

These findings bear relevance for higher-order theories of consciousness because these theories predict a close relationship between conscious experience and metacognition (Carruthers, 2011; Cleeremans, 2011; Lau & Rosenthal, 2011). Therefore, some authors have interpreted dissociations between confidence and visibility as evidence against metacognitive theories of consciousness (Dehaene et al., 2014; Jachs et al., 2015), although it has been argued that higher-order theories are in fact compatible with those dissociations (Rosenthal, 2019). The present study showed that the WEV model, although originally developed to explain confidence and not visibility, provided a good fit to visibility as well, indicating similar statistical regularities of visibility and confidence as a function of stimulus strength and identification accuracy. However, the observation that the statistical properties of confidence and visibility are similar does not necessary imply that their statistical properties are identical. To examine the relation between visibility and confidence more closely, it is necessary to compare these two types of subjective judgments in the same experiment. Experiment 2 was conducted to address this issue.

## Experiment 2

### Methods

Experiment 2 was identical to Experiment 1, except for the differences outlined below.

#### Participants

Thirty-nine participants (four males, 35 females) took part in the experiment. Their age ranged between 18 and 36 years (*M *= 20.8).

#### Experimental task

**Fig. 7:**
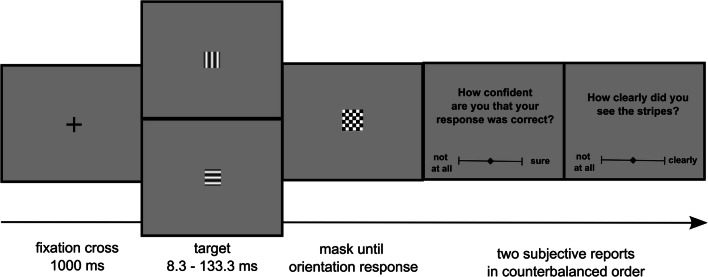
Trial structure of Experiment 2

Figure 7 depicts the time course of one trial. A trial of Experiment 2 was the same as in Experiment 1, except that participants not only reported the subjective visibility of the stimulus but were also asked to report their confidence in having made the correct identification response. For measuring confidence, the question “How confident are you that your response was correct?” appeared on-screen. Participants reported their degree of confidence again using a visual analogue scale and a joystick. The end points were labelled as “not at all” and “sure.” Participants reported visibility and confidence one after the other in the same trial, with the sequence of the two reports counterbalanced across subjects.

#### Design and procedure

The experiment consisted of one training block and nine experimental blocks of 45 trials each. Each of the five SOAs were featured nine times in each block in random order. Participants were instructed to report the orientation of the grating as accurately as possible without time pressure. Moreover, participants were told that their judgments of visibility should be based on their subjective visual experience of the stimulus, and their judgments of confidence should be about their feeling of confidence that their orientation judgment was correct.

### Results

**Fig. 8:**
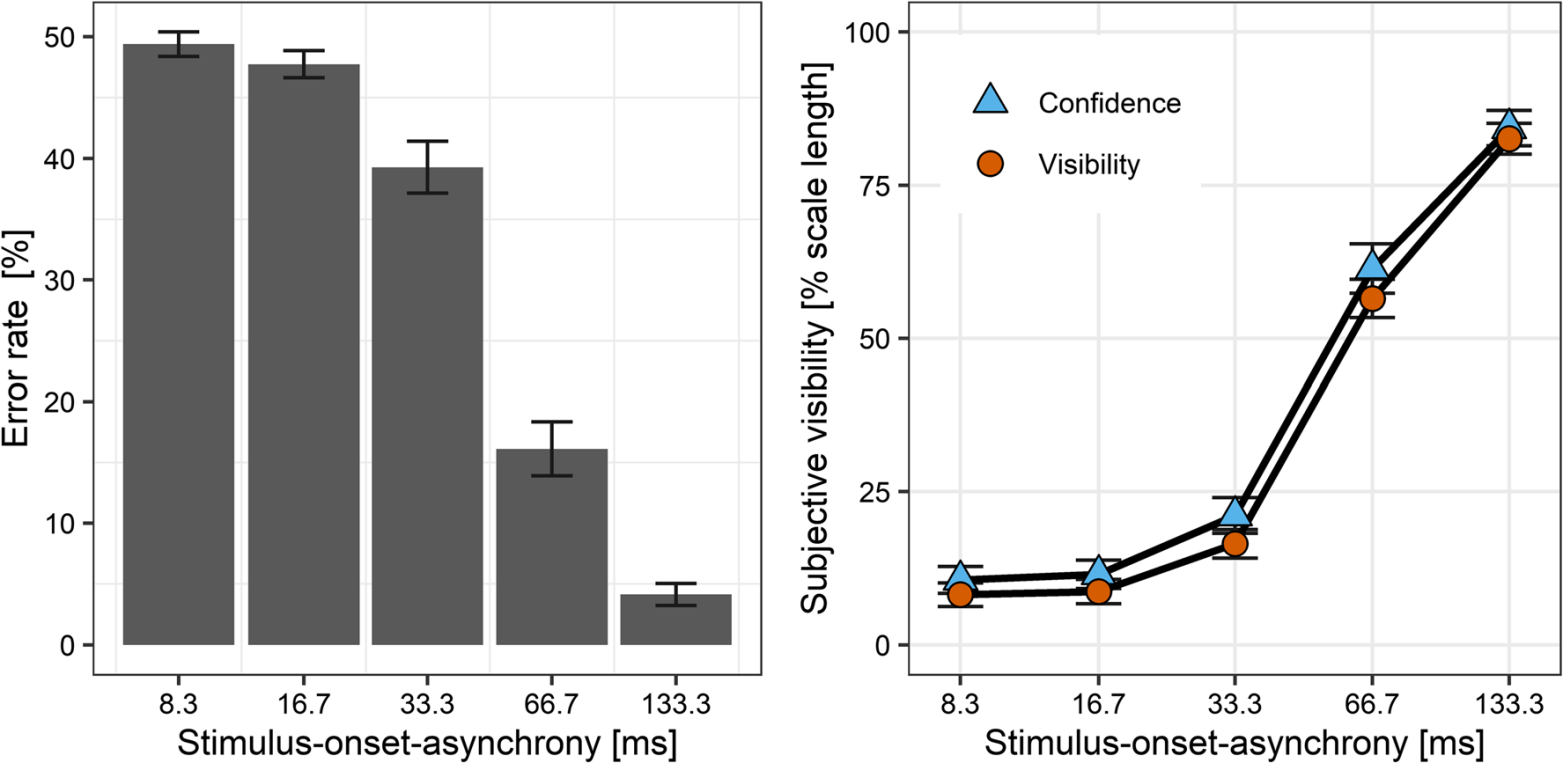
Error rate in the orientation task (left panel) and confidence versus visibility (right panel) as a function of stimulus-onset asynchrony (*x*-axis) in Experiment 2. Bars and symbols indicate observed means. Error bars indicate 1 *SEM*

One participant was excluded from the analysis because her performance was not above chance level, BF_10_ = .13. For the remaining 38 participants, the error rate was at chance level at the SOA of 8.3 ms (*M* = 49.4%, *SD* = 6.2) and very low at the maximum SOA of 133.3 ms (*M* = 4.1%, *SD* = 5.6; see Fig. 8, left panel). Subjective visibility averaged 8.2% (*SD* = 11.8) of the width of the visual analogue scale at the shortest SOA of 8.3 ms and increased to a mean of 82.6% (*SD* = 15.4) at the maximum SOA of 133.3 ms. There was moderate evidence that mean visibility in Experiment 2 was the same as in Experiment 1, BF_10_ = 0.31. Confidence was on average 10.6% (*SD* = 13.5) at the SOA of 8.3 ms and 84.3% (*SD* = 17.6) at the SOA of 133.3 ms (see Fig. 8, right panel). No effect of the order of visibility judgment and confidence judgment was detected on mean error rate BF_10_ = 0.32, mean visibility, BF_10_ = 0.27, or mean confidence, BF_10_ = 0.25. Gamma correlation coefficients between confidence and visibility were large and ranged between M_Γ_ = .64 at the SOA of 66.7 ms and M_Γ_ = .77 at the SOA of 16.7 ms. The evidence was not conclusive whether there was an effect of scale order on gamma correlation coefficients between visibility and confidence for all five SOAs, 0.44 ≤ BF_10_s ≤ 1.1.

#### Model fits

**Fig. 9:**
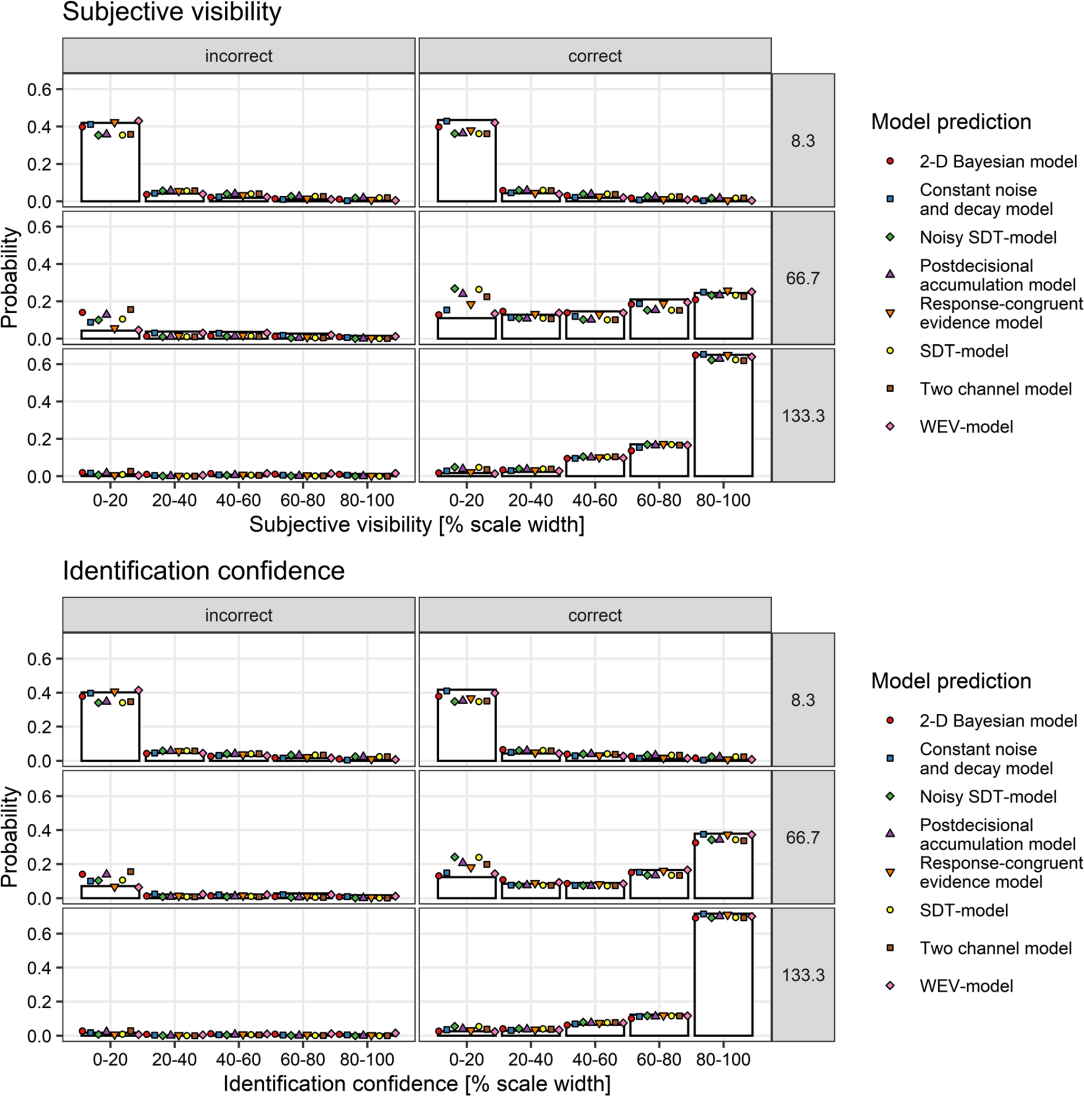
Distribution of subjective visibility (upper panel) and decisional confidence (lower panel) depending on SOA (rows) and accuracy of the response (columns) in Experiment 2. Symbols show the prediction of the different models based on the sets of parameters identified during model fitting

Figure 9 depicts the observed distribution of subjective visibility and identification confidence depending on SOA and accuracy, as well as the predicted distributions from the sets of parameters identified during model fitting assuming constant variances of the decision variable. Replicating Experiment 1, the WEV model appeared to provide a decent account of the distribution of visibility, but the constant noise and decay model seemed to make an accurate prediction as well.

**Fig. 10:**
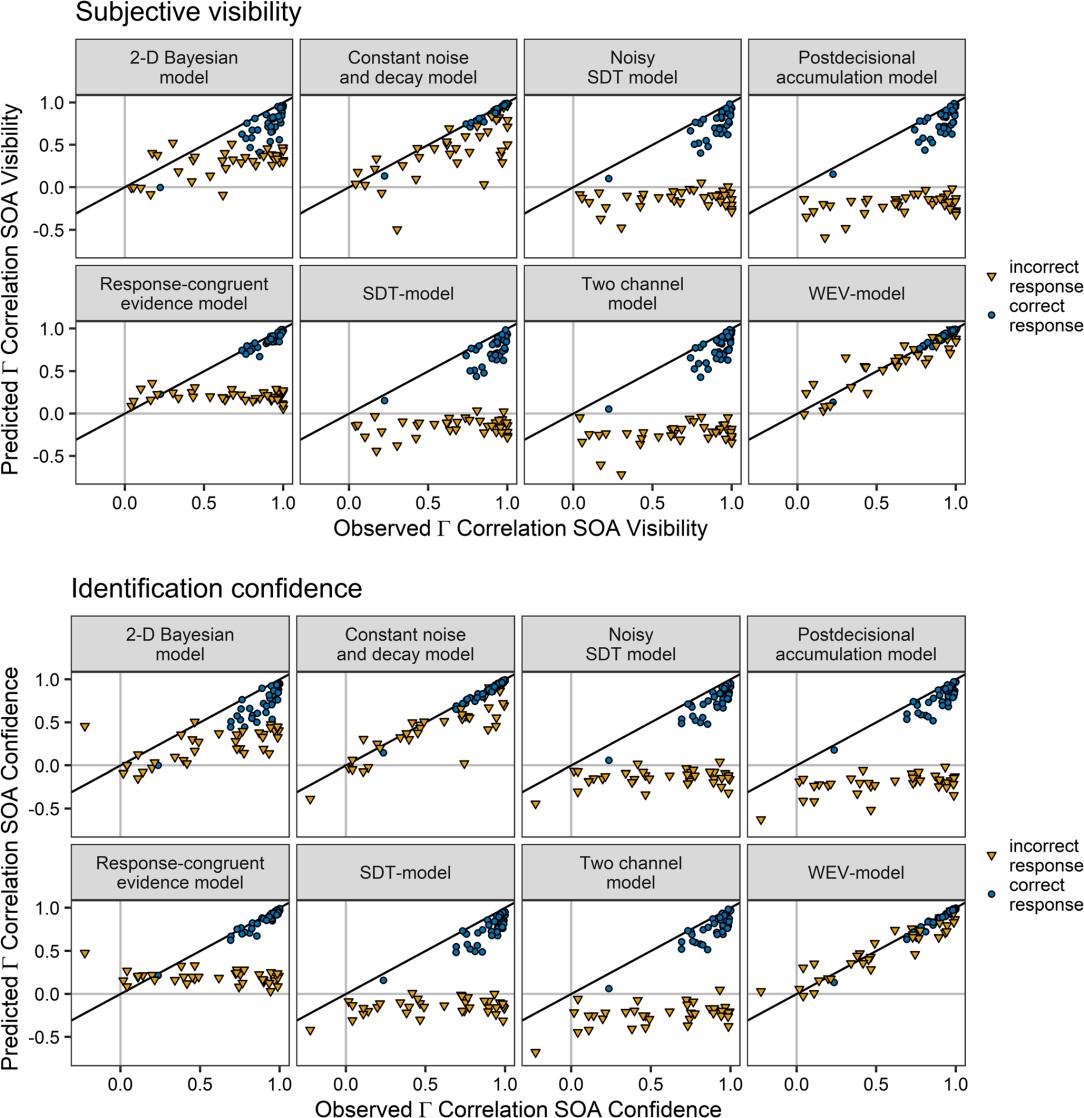
Observed gamma correlation coefficients between SOA and visibility as well as between SOA and confidence vs. gamma correlation coefficients derived from the model fits for subjective visibility (Row 1 and 2) and confidence (Row 3 and 4) for the different models in separate panels and for correct trials (circles) and incorrect trials (triangles). Each symbol represents the data from one participant

Figure 10 shows that the WEV model reproduced the pattern of correlation between both visibility and SOA as well as confidence and SOA. The constant noise and decay model tended to underestimate the correlation between SOA and visibility, but it provided an acceptable account of the correlation between SOA and confidence. The other models did not account for the variability of the correlation between SOA and visibility/confidence in incorrect trials across participants.

#### Formal model comparisons

AIC_c_ and BIC indicated that the WEV model provided the best fit to both visibility and confidence, independently of whether the variances of the decision variables were constant, differed between the two possible identities of the stimulus, or increased with SOA.

**Fig. 11:**
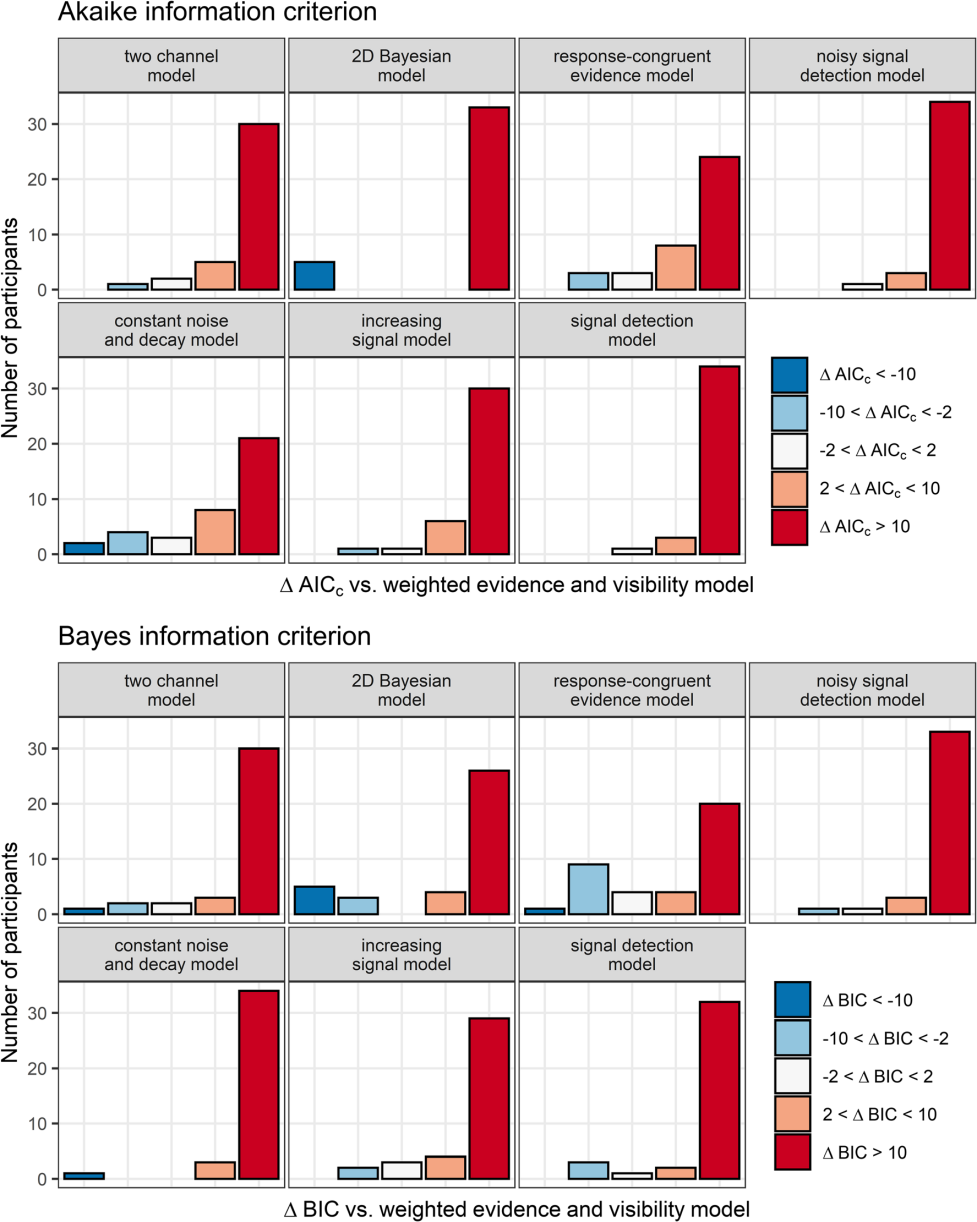
Model fits to subjective visibility. The different panels depict the frequency of AIC_c_- and BIC differences when the WEV model was compared with each of the seven other models assuming constant variances of the decision variable

Concerning visibility and assuming constant variances, Fig. 11 shows that for most participants, the WEV model was associated with substantially smaller AIC_c_ and BIC than each of the other models. Bayes factors indicated that the evidence that the WEV model performed better than the response-congruent evidence model was extremely strong in terms of AIC_c,_ *M*_*ΔAIC*_ = 26.9, 95% HDI [16.5, 35.8], BF_10_ = 9.0×10^3^, and very strong in terms of BIC, *M*_*ΔBIC*_ = 19.2, 95% HDI [9.0, 28.1], BF_10_ = 87.7. For all the other seven models, there was always extremely strong evidence that the WEV performed better in terms of both AIC_c_ and BIC, *M*_*ΔAIC*_s ≥ 16.9, *M*_*ΔBIC*_s ≥ 32.2, BF_10_s = 148.5.

When models were fitted to visibility assuming either different variances associated with the two possible identities of the stimulus or variances increasing with SOA, in both cases, there was extremely strong evidence that the WEV model performed better than the constant noise and decay model and the response-congruent evidence model in terms AIC_c_ and in terms of BIC, *M*_*ΔAIC*_s ≥ 16.9, *M*_*ΔBIC*_s ≥ 24.0, BF_10_s = 342.0.

**Fig. 12:**
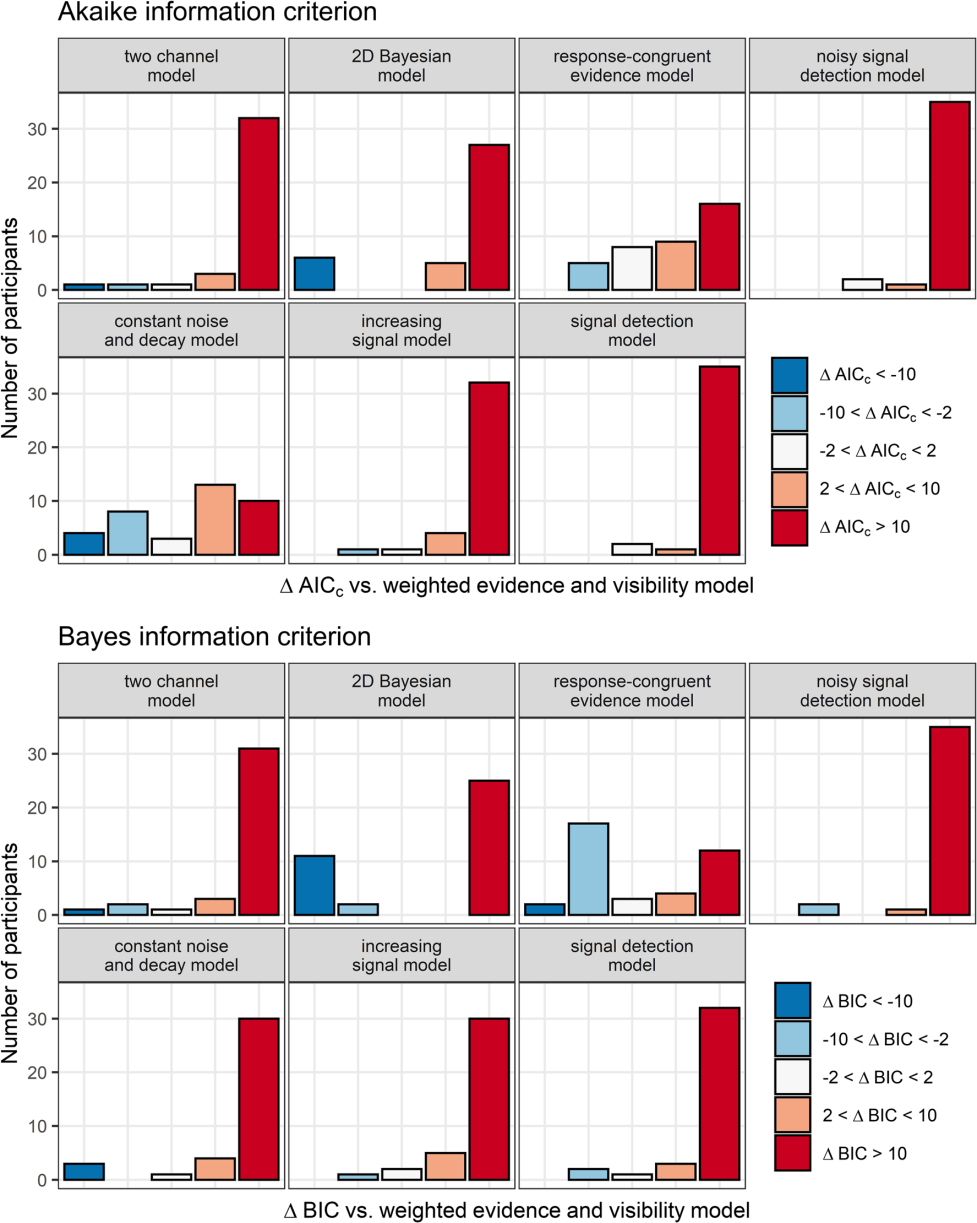
Model fits to identification confidence. The different panels depict the frequency of AIC_c_- and BIC differences when the WEV model was compared with each of the seven other models assuming constant variances of the decision variable

Concerning decision confidence, Fig. 12 shows that while the WEV model again performed best for most comparisons assuming constant variances, there seemed to be only a small advantage compared with the constant noise and decay model in terms of AIC_c_ and no advantage compared with the response-congruent evidence model in terms of BIC. Bayes factors indicated moderate evidence against a difference in model fit between the WEV model and the constant noise and decay model in terms of AIC_c_, *M*_*ΔAIC*_ = 3.2, 95% HDI [−2.3, 8.3], BF_10_ = 0.24, although there was also extremely strong evidence that the WEV model performed better than the constant noise and decay model with respect to BIC, *M*_*ΔBIC*_ = 18.4, 95% HDI [12.5, 23.5], BF_10_ = 2.7×10^5^. Regarding the response-congruent evidence model, the evidence was extremely strong that the WEV model performed better in terms of AIC_c_, M_*ΔAIC*_ = 16.0, 95% HDI [8.7, 22.4], BF_10_ = 575.7, but the evidence was not conclusive with respect to BIC, M_*ΔBIC*_ = 8.3, 95% HDI [1.3, 14.8], BF_10_ = 1.9. Likewise, the evidence in favor of the WEV compared with the 2D Bayesian model was extremely strong in terms of AICc, M_*ΔAIC*_ = 49.6, 95% HDI [24.0, 71.8], BF_10_ = 121.0, but only anecdotal in terms of BIC, M_*ΔAIC*_ = 30.3, 95% HDI [5.4, 52.8], BF_10_ = 2.2. For all the other four models, there was always extremely strong evidence that the WEV performed better in terms of both AIC_c_ and BIC, *M*_*ΔAIC*_s ≥ 71.2, *M*_*ΔBIC*_s ≥ 67.1, BF_10_s ≥ 1.3×10^5^.

When models were fitted to decisional confidence assuming different variances associated with the two possible identities of the stimulus, Bayes factors were not conclusive if the WEV model performed better than the constant noise and decay model in terms of AIC_c_, *M*_*ΔAIC*_ = 6.9, 95% HDI [−0.7, 14.0], BF_10_ = 0.6, but there was extremely strong evidence that the WEV model was better in terms of BIC, *M*_*ΔBIC*_ = 22.2, 95% HDI [14.0, 29.1], BF_10_ = 2.1×10^4^. For the response-congruent evidence model, the evidence was extremely strong that the WEV model performed better in terms of AIC_c_, *M*_*ΔAIC*_ = 27.7, 95% HDI [14.9, 38.7], BF_10_ = 544.3, and strong in terms of BIC, *M*_*ΔBIC*_ = 20.0, 95% HDI [7.5, 31.0], BF_10_ = 16.1.

When models were fitted to decisional confidence assuming that the variances increased as a function of SOA, the evidence that the WEV model performed better than the constant noise and decay model was only moderate in terms of AIC_c_, *M*_*ΔAIC*_ = 5.9, 95% HDI [1.9, 9.3], BF_10_ = 8.8, but extremely strong evidence in terms of BIC, *M*_*ΔBIC*_ = 21.1, 95% HDI [17.0, 24.6], BF_10_ = 5.8×10^10^. For the response-congruent evidence model, the evidence was extremely strong that the WEV model performed better in terms of AIC_c_, *M*_*ΔAIC*_ = 17.6, 95% HDI [9.8, 24.4], BF_10_ = 896.2, and moderate in terms of BIC, *M*_*ΔBIC*_ = 10.0, 95% HDI [2.4, 16.8], BF_10_ = 3.5.

#### Parameter comparisons between visibility and confidence

As the Hessian matrices indicated that the estimates of the parameters of the WEV model with constant variances of the decision variable were less closely correlated with each other on average (*M*_∣*r*∣_ = .11) than the parameters of other two versions of the WEV model (*M*_∣*r*∣_ ′ *s* = .20)*,* the WEV model with constant variances was used to compare parameter sets between visibility and confidence. Descriptive statistics of the parameters of visibility and confidence are found in Supplementary Table S2.

**Fig. 13:**
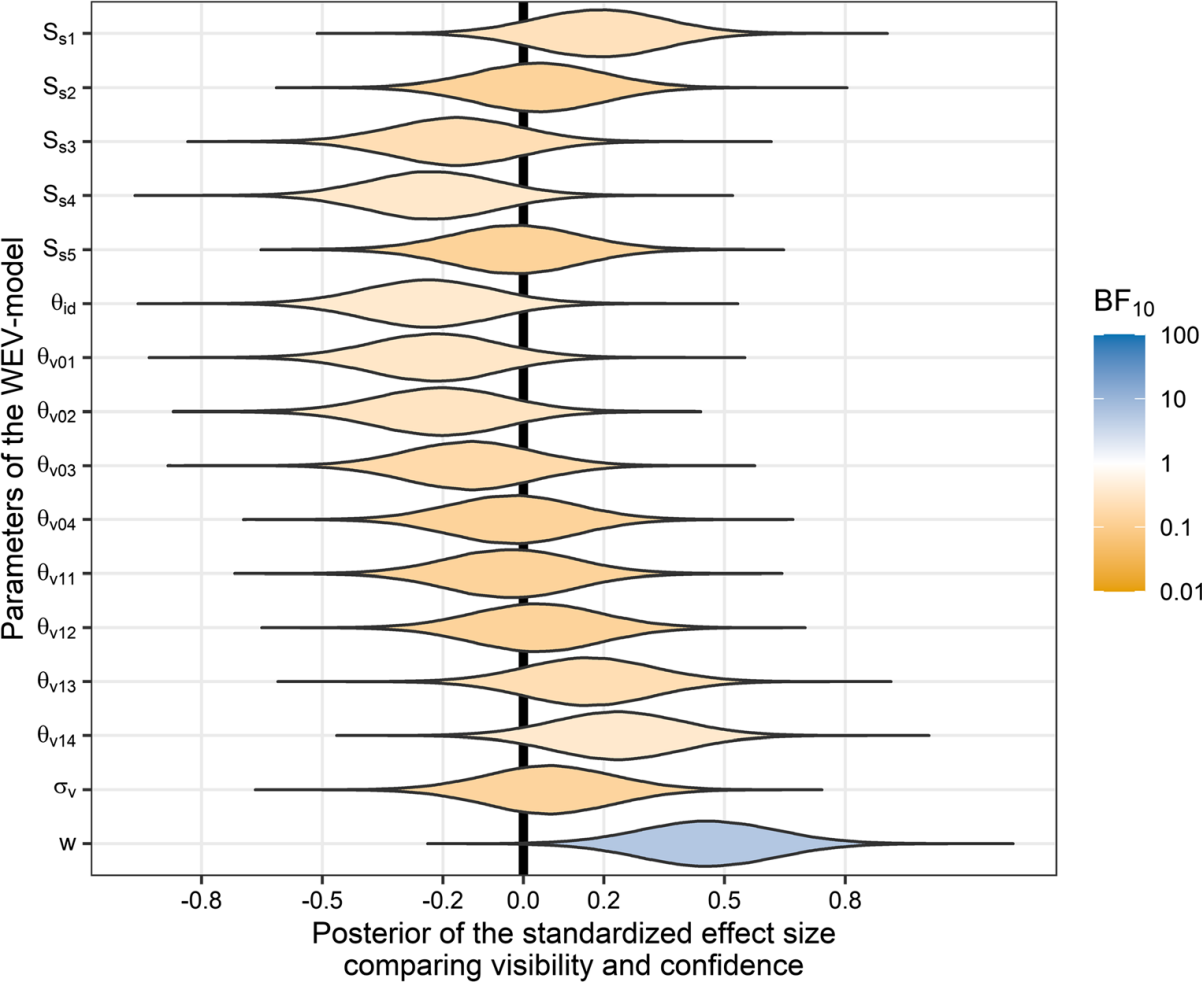
Posterior distributions of the standardized effect size of the comparison between visibility and confidence with respect to each parameter of the WEV model. The standardized effect size is the mean difference between the parameters fitted to visibility and the parameters fitted to confidence, divided by the standard deviation of the difference between the parameters fitted to visibility and the parameters fitted to confidence. Colors indicate the strength of evidence in favor (blue) or against (orange) a difference between visibility and confidence

The correlation between the WEV model parameters obtained when fitting visibility and the parameters obtained when fitting confidence was nearly perfect for all parameters related to the identification judgment, *r*s ≥ .92, and still strong for all parameters related to subjective reports, .58 ≥ all *r*s ≥ .73 (see Supplementary Table S3). Nevertheless, there was moderate evidence for a difference between visibility and confidence with respect to one parameter: The parameter *w,* which captures the weight on evidence about identity-irrelevant features of the stimulus, was greater for visibility than for confidence, *M*_*Δw*_ = .08, 95% HDI [.02 .13], BF_10_ = 5.7. There was evidence that the w-parameter of confidence judgments did not depend on the order of visibility and confidence judgments, *BF*_*10*_ = 0.26; the evidence was not conclusive whether there was an effect of the order of judgments on the *w*-parameter of visibility judgments, BF_10_ = 0.58. For four of the five stimulus strength parameters (i.e., *S*_*s*1_, *S*_*s*2_, *S*_*s*3_, and *S*_*s*5_) for seven out of the eight report criteria (i.e., *θ*_*v*01_, *θ*_*v*02_, *θ*_*v*03_, *θ*_*v*04_, *θ*_*v*11_, *θ*_*v*12_,and *θ*_*v*13_) *a*s well as the noise parameter σ_v_, the Bayes factors indicated moderate evidence against different parameters for visibility and confidence, 0.13 ≤all BF_10_s ≤ 0.32. For one of the stimulus strength parameters *S*_*s*4_, the identification criterion *θ*_*id*_, and one report criterion, *θ*_*v*14_, the evidence for a difference between visibility and confidence was not conclusive, 0.34 ≤all BF_10_s ≤ 0.38. However, the posterior distributions of the standardized effect sizes of the effect of visibility versus confidence suggests that small effects cannot be ruled out for any parameter (see Fig. 13). Finally, there was moderate evidence against a difference between visibility and confidence in terms of model fit of the WEV model, *M*_*ΔAIC*_ = *M*_*ΔBIC*_ = −4.8, 95% HDI [−25.2, 15.9], BF_10_ = 0.14.

Bayes factor comparisons revealed moderate evidence against a difference between the WEV model parameters obtained in Experiment 1 and the parameters fitted to visibility judgments in Experiment 2 for 15 out of 16 parameters, 0.18 ≤ all BF_10_s ≤ 0.33. The only exception was the sensitivity parameter S_s2_, for which the evidence was inconclusive BF_10_ = 0.43 (see Supplementary Fig. S5).

#### Simulating the relationship between confidence and visibility

Given there was only evidence for a difference between confidence and visibility in terms of parameter *w*, we performed two simulations based on the WEV model to examine whether different *w*-parameters are sufficient to explain the relationship between visibility and confidence. To keep all the other parameters identical between confidence and visibility, we first averaged the parameters obtained during the two separate analysis runs for visibility and for confidence except for ***w***. Then, we simulated predictions using these averaged parameters as well as different ***w***-parameters for confidence and visibility.

##### Confidence in absence of visibility and vice versa

**Fig. 14:**
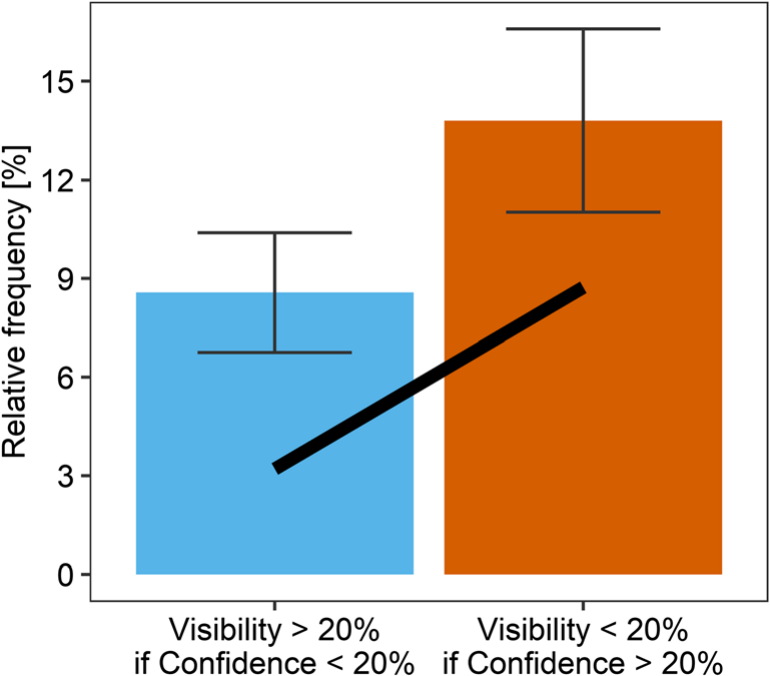
Mean relative frequency of observers reporting a degree of visibility above 20% of the scale width in trials when they reported a degree of confidence below 20% (left), compared with the mean probability of observers reporting a degree of confidence above 20% of the scale width in trials when they reported a degree of visibility below 20% (right). The line indicates the prediction of the WEV model with the assumption that only the *w*-parameter was different between visibility and confidence. Error bars indicate 1 *SEM*

First, it was examined whether the WEV model is able to reproduce the probability of a low degree of visibility or confidence conditioned on each other. Figure 14 shows that visibility and confidence appeared to contradict each other in a moderate number of trials: When observers reported a degree of confidence below 20% of the scale width, they reported a degree of visibility above 20% on average in 8.6% of trials. When observers reported a degree of visibility below 20% of the scale width, they reported a degree of confidence above 20% on average in 13.8% of trials. Figure 14 also shows that the prediction of the WEV model was consistent with the trend that confidence at a low degree of visibility occurred more often than visibility at a low level of confidence. Still, it can also be seen that the model tended to underestimate the frequency of a higher degree of visibility in trials with a minimal degree of confidence, as well as the frequency of a higher degree of confidence in trials with a minimal degree of visibility.

##### Correlation between visibility and confidence

Finally, we examined whether assuming different *w*-parameters is sufficient to explain the correlation between visibility and confidence. However, the correlation between visibility and confidence implied by the WEV model and only distinct *w*-parameters is far greater than the correlation observed empirically (see Supplementary Fig. S6). In addition, the variability across participants with respect to the correlation between confidence and visibility was not recovered by the prediction.

### Discussion

Experiment 2 replicated Experiment 1, as the best fit to visibility judgments in a postmasked orientation task was provided by the WEV model. It also replicated previous studies in showing that the WEV model provides a better account of confidence in binary perceptual decisions than the SDT model, the noisy SDT model, the postdecisional accumulation model, and the two-channel model (Rausch et al., 2018, 2020). Experiment 2 also provided some evidence that the WEV model is a better explanation of confidence than the constant noise and decay model, the response-congruent evidence model, as well as the 2D Bayesian model, although in the present study, a benefit of the WEV model was corroborated by only one out of two goodness-of-fit measures for each model.

The question arises whether the results in Experiment 2 are specific to the present paradigm requiring participants to report both visibility and confidence in each single trial. There might have been a mutual influence of the two judgments, especially as visibility and confidence were closely correlated. Moreover, the instruction to rate both visibility and confidence after each single trial could also have let participants to rate visibility and confidence as dissimilar as possible. However, there is no evidence that the present design of requiring subjects to rate both visibility and confidence had any effect on the data: In terms of model fits, the WEV model provided the best fit to visibility both when there was no confidence judgment in Experiment 1, and when there was one in Experiment 2. The WEV model had also consistently provided the best fit in previous studies using only confidence judgments and the same stimuli as the present study (Rausch et al., 2018; Rausch et al., 2020). In terms of parameters, we detected no systematic differences between the parameter sets fitted to visibility in Experiments 1 and 2. If participants in Experiment 2 had systematically shifted their criteria in an attempt to decorrelate visibility and confidence, we would have expected a difference in rating criteria between Experiments 1 and 2, but this was not the case. If observers had shifted their criteria unsystematically to decorrelate visibility and confidence, the resulting unsystematic variability should have affected σ_v_, which reflects all variability that is independent from the noise stemming from the identification judgment. Overall, any mutual influence of visibility and confidence judgments was moderate at best.

What does Experiment 2 imply with respect to the three different hypotheses about the distinction between confidence and visibility? According to the feature hypothesis, visibility judgments depend more strongly on sensory evidence about identity-irrelevant features than confidence judgments do. Consistent with the feature hypothesis, the weight parameter *w*, which assesses the relative amount of influence of sensory evidence about identity-irrelevant features of the stimulus compared with the influence of sensory evidence about the identity, was greater for visibility than for confidence. This view is a modification of an earlier account, where we proposed that when participants report their confidence, they evaluate only those stimulus characteristics relevant for task (i.e., the orientation of the stimulus; Rausch et al., 2015; Rausch & Zehetleitner, 2016). However, previous studies indicated (Navajas et al., 2017; Rausch et al., 2018) and Experiment 2 corroborated the view that sensory evidence about identity-irrelevant features of the stimulus are involved in the calculation of decisional confidence, too. Thus, the distinction between confidence and visibility in the present study appears to lie in the relative amount of influence of sensory evidence about identity-irrelevant features.

Concerning the metacognitive hypothesis (Charles et al., 2013; Jachs et al., 2015; Overgaard & Sandberg, 2012), it would have been expected that confidence and visibility had been associated with different *σ*_*v*_ parameters. The reason is that *σ*_*v*_ is sensitive to noise generated by metacognitive processes. If visibility and confidence rely in part on different metacognitive processes, and if these two metacognitive systems are not completely on par with each other in terms of noise, visibility and confidence are expected be associated with different *σ*_*v*_ parameters. However, the Bayesian analysis revealed some evidence that the *σ*_*v*_ parameters estimated from visibility judgments and *σ*_*v*_ parameters estimated from confidence judgments were the same. Still, while there was no evidence for metacognitive processes specific to confidence in the present study, it is still possible that those processes can be detected in more sensitive paradigms. For example, it can be speculated that experimental stimuli designed to dissociate confidence and/or visibility from identification accuracy (Koizumi et al., 2015; Maniscalco & Lau, 2016; Odegaard et al., 2018; Samaha et al., 2016) may be more apt to separate the processes underlying confidence and visibility and could be able to detect metacognitive processes specific to decision confidence in future studies.

Concerning the criterion hypothesis (Wierzchoń et al., 2012), the present study suggested that seven out of eight criteria are shared between visibility and confidence, and the evidence about the eighth criterion was inconclusive. Consequently, the distinction between visibility and confidence, at least in the present postmasked orientation discrimination task, cannot be explained by participants’ applying different sets of criteria on the same decision variable. Yet it should be noted that it is reasonable to assume that other sets of criteria are adopted in other tasks.

Visibility and confidence in the present study were strongly correlated. Nevertheless, participants occasionally reported some of confidence in absence of visibility and visibility in absence of confidence. The probabilities of confidence in absence of visibility and vice versa were only partially explained by the WEV model under the assumption of different *w*-parameters. On the one hand, the model correctly predicted that observers reported some confidence in being correct, although they reported the visibility of the stimulus was low more frequently than the opposite pattern of reports (i.e., observers reported less frequently some visibility of the stimulus, although they were minimally confident about their orientation judgment). The existence of confidence in the absence of visibility is consistent with previous studies (Charles et al., 2013; Jachs et al., 2015; Rausch & Zehetleitner, 2016; Zehetleitner & Rausch, 2013). According to the WEV model, confidence without subjective visibility is possible when observer obtain some evidence about the identity at a short SOA. At short SOAs, the average strength of evidence about identity-irrelevant features will be low, which causes observers to make more conservative subjective reports. As the weight on identity-irrelevant features is greater for visibility than for confidence, identity-irrelevant evidence causes observers more often to report no visibility at all than to report no confidence. On the other hand, visibility in absence of confidence and confidence in absence of visibility occurred more often than they should according to the WEV model. The existence of visibility in absence of confidence has also been reported previously (Sandberg et al., 2010). In addition, the model overestimated the correlation between visibility and confidence. At a consequence, it seems that a complete generative model of confidence and visibility judgments may require independent sources of noise for both types of judgments, which may stem from the two subsequent motor responses. Overall, it appears that further research is necessary to comprehensively describe the decision mechanisms underlying visibility and decision confidence.

## General discussion

The present study suggested that the WEV model can be generalized to subjective visibility. The WEV model provided a better account of visibility judgments in a postmasked orientation identification task than did the standard SDT rating model, the noisy SDT model, the postdecisional accumulation model, the two-channel model, the constant noise and decay model, and the response-congruent evidence model. Moreover, estimating the parameters of the WEV model revealed that observers relied more strongly on sensory evidence about stimulus strength when they reported their subjective degree of visibility, compared with when they reported their confidence about the correctness of the identification judgment.

### Does subjective visibility depend on metacognition?

The present study bears implications for theories of consciousness. One group of theories referred to as higher-order theories of consciousness posit a close relation between visual experience and metacognition (Carruthers, 2011; Cleeremans, 2011; Lau & Rosenthal, 2011; Shea & Frith, 2019). Subjective confidence is often considered to be a hallmark of metacognition (Kepecs & Mainen, 2012). Therefore, some authors have interpreted dissociations between confidence and visibility as evidence against metacognitive theories of consciousness (Dehaene et al., 2014; Jachs et al., 2015), although others have argued that confidence judgments should not be used to test higher-order theories (Rosenthal, 2019).

The present study is consistent with the view that confidence and visibility are generated by the same or similar mechanisms: First, no qualitative differences in the statistical pattern underlying confidence and visibility were observed. The WEV model, which had been developed to account for decisional confidence (Rausch et al., 2018), was also able to explain visibility. This means that confidence and visibility are not only related to the identification decision, but both of them also require a process sensitive to the reliability of the percept based on decision-irrelevant features of the stimulus. Second, Experiment 2 showed evidence against a difference between confidence and visibility with respect to the parameter *σ*_*v*_. This parameter quantifies the amount of unsystematic noise present in the subjective report, but not within the identification judgment, and thus is sensitive to the amount insight into one’s own decision. The fact that visibility and confidence are associated with the same or a similar σ_v_ implies that the underlying metacognitive mechanisms are at least similar.

Yet the present study is also inconsistent with the view that confidence and visibility are identical or interchangeable (Lau & Rosenthal, 2011; Seth et al., 2008). The reason is that observers seem to apply different weight on sensory evidence about the identity of the stimulus and sensory evidence irrelevant to the identity: Observers relied to a greater extent on evidence parallel to the identity of the stimulus in judgments about visibility compared with judgments about confidence. A possible interpretation for this finding is that one system is capable of making two related but different kinds of judgments. Confidence can be thought of as an observer’s judgment about the probability of being correct. By contrast, visibility judgments may reflect an observer’s judgment about the estimated quality of the visual representation. Depending on the kind of judgment the observer intends to make, the observer weights the available information in different ways.

### Is subjective visibility all-or-nothing or gradual?

A second controversial topic is whether visual awareness is all-or-nothing, or gradual. The present study may contribute to this debate because it is shown that a model assuming only continuous decision variables provides an adequate description of visibility judgments. This observation is consistent with the view that subjective visibility is in fact gradual. The debate is relevant for theories about consciousness because global workspace theory asserts that awareness of conscious contents is binary (Dehaene et al., 2003; Sergent & Dehaene, 2004). Evidence for all-or-nothing conscious perception stems from the presentation of words during the attentional blink (Sergent & Dehaene, 2004) and from a masked numerical discrimination task (Del Cul et al., 2007). In both studies, visibility judgments were strongly concentrated at the scale ends, and medium levels of subjective visibility were (mostly) absent. In contrast, medium levels of subjective visibility were observed with masked words (Sergent & Dehaene, 2004), masked geometrical shapes (Sandberg et al., 2010; Sandberg et al., 2011), characters during the attentional blink (Nieuwenhuis & Kleijn, 2011), random dot kinematograms (Rausch & Zehetleitner, 2014), and low-contrast gratings (Rausch & Zehetleitner, 2016). In addition, a follow-up study did not replicate the absence of medium levels of visibility of words during the attentional blink (Nieuwenhuis & Kleijn, 2011). Windey et al. (2013) proposed that these contradictory findings can be reconciled by consideration of the depth of stimulus processing required by the task: When observers performed the numerical identification task, visibility of identical stimuli increased more abruptly as when observers performed a color identification task. Likewise, visibility in a masked word identification task was more strongly concentrated at the scale ends than visibility in a color identification task (Windey et al., 2014), and medium levels of visibility occurred more frequently in a same-or-different task about the physical features of the stimulus than when the task involved a semantic comparison (Anzulewicz et al., 2015).

The present study suggests that the WEV model, which assumes only continuous inner variables, provides an adequate account for the distribution of subjective visibility even though subjective visibility was concentrated more on the scale ends than on the scale center in the present study. This observation is important because it implies that even more abrupt transitions between no visibility and full visibility as a function of stimulus strength are consistent with gradual internal states. At a consequence, instead of interpreting the distributions of subjective visibility qualitatively, it may be more apt to use mathematical modelling to assess whether the distribution of subjective visibility is consistent with continuous inner states (e.g., Ricker et al., 2017; Swagman et al., 2015).

### Implications for future studies using visibility judgments

The present study may have two implications for future studies: First, with respect to those studies whose aim is to measure perceptual sensitivity or metacognition, it is implied that researchers should critically evaluate whether it is adequate to apply standard signal detection theory (Green & Swets, 1966; Macmillan & Creelman, 2005; Wickens, 2002) or Type 2 signal detection theory (Galvin et al., 2003; Kunimoto et al., 2001; Maniscalco & Lau, 2012, 2014). The reason is that standard signal detection theory provided a poor fit to visibility and confidence in the present study. While it is considered good practice to assess whether the distributions of evidence are consistent with Gaussian distributions (Macmillan & Creelman, 2005), researchers often do not test whether signal detection theory in general is adequate to account for their data. Up to now, it is unknown whether there are adverse consequences if measures of perceptual sensitivity from Type 1 SDT and measures of metacognition from Type 2 signal detection theory are applied to data generated according to the WEV model. A systematic investigation is pending whether these measures are robust to contamination from identity-irrelevant sensory evidence, which is assumed by the WEV model.

Second, with respect to those studies that aim to identify the generative model underlying visibility judgments, the present study implies that a complete model of visibility does not only involve representations about the feature relevant to the identification judgments but also information about stimulus strength that is partially independent from the information about the identity of the stimulus. That feature of the WEV model is not included in other models of decision confidence (Aitchison et al., 2015; Green & Swets, 1966; Maniscalco & Lau, 2016; Moran et al., 2015; Peters et al., 2017; Pleskac & Busemeyer, 2010; Ratcliff & Starns, 2009, 2013; Rausch & Zehetleitner, 2017).

However, there are also several reasons why the true generative model is probably even more complex than the WEV model: First, the present analysis modelled confidence and visibility judgments in two separate runs of analysis. However, this approach turned out to be insufficient to account for the correlation between confidence and visibility. For a complete model of confidence and visibility, future studies may find it necessary to explicitly model the joint distribution of confidence and visibility judgments, because such a modelling analysis may be able to distinguish between shared and independent noise in visibility and confidence judgments, respectively. Second, the WEV model is silent about the dynamics of the decision process, and although the correlations between visibility and reaction times in the present study were weak at best, it has been argued that a complete generative model of visibility judgments should explain the timing of decisions and subjective reports as well (Jachs et al., 2015). Finally, the WEV model only applies to experiments where one dimension of stimulus strength is varied, but recent studies showed that by varying signal strength and signal-to-noise-ratio, confidence can be changed without changing identification accuracy (Koizumi et al., 2015; Odegaard et al., 2018; Samaha et al., 2016). To accommodate those effects, the WEV model would have to be extended again. Overall, future studies seem necessary to identify the generative model of visibility judgments.

### Conclusion

Subjective visibility, the degree to which observers are consciously seeing the stimulus, is best explained by the recent weighted evidence and visibility model, according to which visibility judgments depend on evidence relevant to the identification of a stimulus as well as evidence irrelevant to the identity of the stimulus. The standard signal detection model, the noisy SDT model, the postdecisional accumulation model, the two-channel model, the constant noise and decay model, the response-congruent evidence model, and the two-dimensional Bayesian model all fail to account for the statistical properties of visibility judgments.

## Supplementary Information

ESM 1 (PDF 787 kb)
