# Supplementary material for: Modelling visibility judgments using models of decision confidence
Source: Atten Percept Psychophys. 2021 Jun 4;83(8):3311–36. doi: 10.3758/s13414-021-02284-3 (PMC8550028; doi:10.3758/s13414-021-02284-3)
Supplement: Supplementary file 1 — (PDF 787 kb) [file 13414_2021_2284_MOESM1_ESM.pdf]

Supplementary material to *Modelling visibility judgments using models of decision confidence*

Manuel Rausch, Sebastian Hellmann, and Michael Zehetleitner

Catholic University of Eichstätt-Ingolstadt

Author Note

Manuel Rausch, Katholische Universität Eichstätt-Ingolstadt, Philosophisch-pädagogische Fakultät, Fachgebiet Psychologie II, Eichstätt, Germany; Sebastian Hellmann, Katholische Universität Eichstätt-Ingolstadt, Philosophisch-pädagogische Fakultät, Fachgebiet Psychologie II, Eichstätt, Germany; Michael Zehetleitner, Katholische Universität Eichstätt-Ingolstadt, Philosophisch-pädagogische Fakultät, Fachgebiet Psychologie II, Eichstätt, Germany.

This research was in parts supported by the Deutsche Forschungsgemeinschaft (grant numbers ZE 887/8-1, RA2988/3-1). The funders had no role in study design, data collection, analysis, decision to publish, or preparation of the manuscript.

Correspondence should be addressed to: Manuel Rausch, Katholische Univerität Eichstätt-Ingolstadt. Psychologie II, Ostenstraße 25, 85072 Eichstätt, Germany. E-mail: manuel.rausch@ku.de. Phone: +49 8421 93 21639.

Supplementary Table S1. Formulae for calculating the likelihood of the data for each of the models depending on the identity of the stimulus S_{id}, the stimulus strength as a model parameter S_s, the physical strength, i.e. the SOA Δt, the identification judgment R_{id}, as well as the visibility judgment R_v. ϕ indicates the Gaussian probability density function.

Model	R_{id}	R_v	$P(R_{id}, R_v \vert S_{id}, S_s)$
SDT	-1	1	$\int_{\theta_{v01}}^{\theta_{id}} \phi(x \vert \frac{1}{2} \times S_{id} \times S_s, \sigma_{id})\ dx$
	-1	2	$\int_{\theta_{v02}}^{\theta_{v01}} \phi(x \vert \frac{1}{2} \times S_{id} \times S_s, \sigma_{id})\ dx$
	-1	5	$\int_{-\infty}^{\theta_{v04}} \phi(x \vert \frac{1}{2} \times S_{id} \times S_s, \sigma_{id})\ dx$

	1	1	$\int_{\theta_{id}}^{\theta_{v11}} \phi(x \vert \frac{1}{2} \times S_{id} \times S_s, \sigma_{id})\ dx$
	1	2	$\int_{\theta_{v11}}^{\theta_{v12}} \phi(x \vert \frac{1}{2} \times S_{id} \times S_s, \sigma_{id})\ dx$
	1	5	$\int_{\theta_{v14}}^{\infty} \phi(x \vert \frac{1}{2} \times S_{id} \times S_s, \sigma_{id})\ dx$
Noisy SDT	-1	1	$\int_{-\infty}^{\theta_{id}} \phi\left(x \middle\vert \frac{1}{2} \times S_{id} \times S_s, \sigma_{id}\right) \times \left(\int_{\theta_{v01}}^{\infty} \phi(y \vert x, \sigma_v) dy\right) dx$
	-1	2	$\int_{-\infty}^{\theta_{id}} \phi\left(x \middle\vert \frac{1}{2} \times S_{id} \times S_s, \sigma_{id}\right) \times \left(\int_{\theta_{v02}}^{\theta_{v01}} \phi(y \vert x, \sigma_v) dy\right) dx$
	-1	5	$\int_{-\infty}^{\theta_{id}} \phi\left(x \middle\vert \frac{1}{2} \times S_{id} \times S_s, \sigma_{id}\right) \times \left(\int_{-\infty}^{\theta_{v04}} \phi(y \vert x, \sigma_v) dy\right) dx$

	1	1	$\int_{\theta_{id}}^{\infty} \phi\left(x \middle\| \frac{1}{2} \times S_{id} \times S_s, \sigma_{id}\right) \times \left(\int_{-\infty}^{\theta_{v11}} \phi(y \mid x, \sigma_v) dy\right) dx$
	1	2	$\int_{\theta_{id}}^{\infty} \phi\left(x \middle\| \frac{1}{2} \times S_{id} \times S_s, \sigma_{id}\right) \times \left(\int_{\theta_{v11}}^{\theta_{v12}} \phi(y \mid x, \sigma_v) dy\right) dx$
	1	5	$\int_{\theta_{id}}^{\infty} \phi\left(x \middle\| \frac{1}{2} \times S_{id} \times S_s, \sigma_{id}\right) \times \left(\int_{\theta_{v14}}^{\infty} \phi(y \mid x, \sigma_v) dy\right) dx$
Constant noise and decay model	-1	1	$\int_{-\infty}^{\theta_{id}} \phi\left(x \middle\| \frac{1}{2} \times S_{id} \times S_s, \sigma_{id}\right) \times \left(\int_{\theta_{\mathrm{v}01}}^{\infty} \phi(y \mid x \times \rho_S, \sigma_v) dy\right) dx$
	-1	2	$\int_{-\infty}^{\theta_{id}} \phi\left(x \middle\| \frac{1}{2} \times S_{id} \times S_s, \sigma_{id}\right) \times \left(\int_{\theta_{\mathrm{v}02}}^{\theta_{\mathrm{v}01}} \phi(y \mid x \times \rho_S, \sigma_v) dy\right) dx$
	-1	5	$\int_{-\infty}^{\theta_{id}} \phi\left(x \middle\| \frac{1}{2} \times S_{id} \times S_s, \sigma_{id}\right) \times \left(\int_{-\infty}^{\theta_{\mathrm{v}04}} \phi(y \mid x \times \rho_S, \sigma_v) dy\right) dx$

	1	1	$$\int_{\theta_{id}}^{\infty} \phi\left(x \middle\| \frac{1}{2} \times S_{id} \times S_s, \sigma_{id}\right) \times \left(\int_{-\infty}^{\theta_{v11}} \phi(y \mid x \times \rho_S, \sigma_v) dy\right) dx$$
	1	2	$$\int_{\theta_{id}}^{\infty} \phi\left(x \middle\| \frac{1}{2} \times S_{id} \times S_s, \sigma_{id}\right) \times \left(\int_{\theta_{v11}}^{\theta_{v12}} \phi(y \mid x \times \rho_S, \sigma_v) dy\right) dx$$
	1	5	$$\int_{\theta_{id}}^{\infty} \phi\left(x \middle\| \frac{1}{2} \times S_{id} \times S_s, \sigma_{id}\right) \times \left(\int_{\theta_{v14}}^{\infty} \phi(y \mid x \times \rho_S, \sigma_v) dy\right) dx$$
WEV-model	-1	1	$$\int_{-\infty}^{\theta_{id}} \phi\left(x \middle\| \frac{1}{2} \times S_{id} \times S_s, \sigma_{id}\right) \times \left(\int_{\theta_{\mathrm{v}01}}^{\infty} \phi(y \mid (1-w) \times x + w \times R_{id} \times (S_s - \bar{S_s}), \sigma_v) dy\right) dx$$
	-1	2	$$\int_{-\infty}^{\theta_{id}} \phi\left(x \middle\| \frac{1}{2} \times S_{id} \times S_s, \sigma_{id}\right) \times \left(\int_{\theta_{\mathrm{v}02}}^{\theta_{\mathrm{v}01}} \phi(y \mid (1-w) \times x + w \times R_{id} \times (S_s - \bar{S_s}), \sigma_v) dy\right) dx$$
	-1	5	$$\int_{-\infty}^{\theta_{id}} \phi\left(x \middle\| \frac{1}{2} \times S_{id} \times S_s, \sigma_{id}\right) \times \left(\int_{-\infty}^{\theta_{\mathrm{v}04}} \phi(y \mid (1-w) \times x + w \times R_{id} \times (S_s - \bar{S_s}), \sigma_v) dy\right) dx$$

	1	1	$\int_{\theta_{id}}^{\infty} \phi\left(x \middle| \frac{1}{2} \times S_{id} \times S_s, \sigma_{id}\right) \times \left(\int_{-\infty}^{\theta_{v11}} \phi(y \mid (1-w) \times x + w \times R_{id} \times (S_s - \overline{S_s}), \sigma_v) dy\right) dx$
	1	2	$\int_{\theta_{id}}^{\infty} \phi\left(x \middle| \frac{1}{2} \times S_{id} \times S_s, \sigma_{id}\right) \times \left(\int_{\theta_{c11}}^{\theta_{v12}} \phi(y \mid (1-w) \times x + w \times R_{id} \times (S_s - \overline{S_s}), \sigma_v) dy\right) dx$
	1	5	$\int_{\theta_{id}}^{\infty} \phi\left(x \middle| \frac{1}{2} \times S_{id} \times S_s, \sigma_{id}\right) \times \left(\int_{\theta_{v14}}^{\infty} \phi(y \mid (1-w) \times x + w \times R_{id} \times (S_s - \overline{S_s}), \sigma_v) dy\right) dx$
Two-channel model	-1	1	$\int_{-\infty}^{\theta_{id}} \phi\left(x \middle| \frac{1}{2} \times S_{id} \times S_s, \sigma_{id}\right) dx \times \int_{\theta_{\mathrm{v}01}}^{\infty} \phi(y \mid \frac{1}{2} \times S_{id} \times S_s \times a, 1) dy$
	-1	2	$\int_{-\infty}^{\theta_{id}} \phi\left(x \middle| \frac{1}{2} \times S_{id} \times S_s, \sigma_{id}\right) dx \times \int_{\theta_{\mathrm{v}02}}^{\theta_{\mathrm{v}01}} \phi(y \mid \frac{1}{2} \times S_{id} \times S_s \times a, 1) dy$
	-1	5	$\int_{-\infty}^{\theta_{id}} \phi\left(x \middle| \frac{1}{2} \times S_{id} \times S_s, \sigma_{id}\right) dx \times \int_{-\infty}^{\theta_{\mathrm{v}04}} \phi(y \mid \frac{1}{2} \times S_{id} \times S_s \times a, 1) dy$

	1	1	$\int_{\theta_{id}}^{\infty} \phi\left(x \middle\| \frac{1}{2} \times S_{id} \times S_s, \sigma_{id}\right) dx \times \int_{-\infty}^{\theta_{v11}} \phi(y \mid \frac{1}{2} \times S_{id} \times S_s \times a, 1) dy$
	1	2	$\int_{\theta_{id}}^{\infty} \phi\left(x \middle\| \frac{1}{2} \times S_{id} \times S_s, \sigma_{id}\right) dx \times \int_{\theta_{v11}}^{\theta_{v12}} \phi(y \mid \frac{1}{2} \times S_{id} \times S_s \times a, 1) dy$
	1	5	$\int_{\theta_{id}}^{\infty} \phi\left(x \middle\| \frac{1}{2} \times S_{id} \times S_s, \sigma_{id}\right) dx \times \int_{\theta_{v14}}^{\infty} \phi(y \mid \frac{1}{2} \times S_{id} \times S_s \times a, 1) dy$
Postdecisional accumulation model	-1	1	$\int_{-\infty}^{\theta_{id}} \phi\left(x \middle\| \frac{1}{2} \times S_{id} \times S_s, \sigma_{id}\right) \times \left(\int_{\theta_{v01}}^{\infty} \phi(y \mid x + S_{id} \times S_s \times b, \sqrt{b}) dy \right) dx$
	-1	2	$\int_{-\infty}^{\theta_{id}} \phi\left(x \middle\| \frac{1}{2} \times S_{id} \times S_s, \sigma_{id}\right) \times \left(\int_{\theta_{v02}}^{\theta_{v01}} \phi(y \mid x + S_{id} \times S_s \times b, \sqrt{b}) dy \right) dx$
	-1	5	$\int_{-\infty}^{\theta_{id}} \phi\left(x \middle\| \frac{1}{2} \times S_{id} \times S_s, \sigma_{id}\right) \times \left(\int_{-\infty}^{\theta_{v04}} \phi(y \mid x + S_{id} \times S_s \times b, \sqrt{b}) dy \right) dx$

	1	1	$\int_{\theta_{id}}^{\infty} \phi\left(x\middle\|\frac{1}{2}\times S_{id}\times S_s,\sigma_{id}\right)\times\left(\int_{-\infty}^{\theta_{v11}} \phi(y\ \|x\ +\ S_{id}\times S_s\times b,\sqrt{b})dy\right)dx$
	1	2	$\int_{\theta_{id}}^{\infty} \phi\left(x\middle\|\frac{1}{2}\times S_{id}\times S_s,\sigma_{id}\right)\times\left(\int_{\theta_{v11}}^{\theta_{v12}} \phi(y\ \|x\ +\ S_{id}\times S_s\times b,\sqrt{b})dy\right)dx$
	1	5	$\int_{\theta_{id}}^{\infty} \phi\left(x\middle\|\frac{1}{2}\times S_{id}\times S_s,\sigma_{id}\right)\times\left(\int_{\theta_{v14}}^{\infty} \phi(y\ \|x\ +\ S_{id}\times S_s\times b,\sqrt{b})dy\right)dx$
Response-congruent evidence model	-1	1	$\int_{-\infty}^{\theta_{v01}} \phi\left(x\|\frac{1}{2}(1-\ S_{id})\times S_s-\theta_{id},\sigma_{id}\right)\times\left(\int_{-\infty}^{x} \phi(y\|\frac{1}{2}(S_{id}-1)\times S_s+\theta_{id},\sigma_{id})\,dy\right)dx$
	-1	2	$\int_{\theta_{c01}}^{\theta_{v02}} \phi\left(x\|\frac{1}{2}(1-\ S_{id})\times S_s-\theta_{id},\sigma_{id}\right)\times\left(\int_{-\infty}^{x} \phi(y\|\frac{1}{2}(S_{id}-1)\times S_s+\theta_{id},\sigma_{id})\,dy\right)dx$
	-1	5	$\int_{\theta_{v04}}^{\infty} \phi\left(x\|\frac{1}{2}(1-\ S_{id})\times S_s-\theta_{id},\sigma_{id}\right)\times\left(\int_{-\infty}^{x} \phi(y\|\frac{1}{2}(S_{id}-1)\times S_s+\theta_{id},\sigma_{id})\,dy\right)dx$

	1	1	$\int_{-\infty}^{\theta_{v11}} \phi\left(x\mid\frac{1}{2}(S_{id}-1)\times S_s+\theta_{id},\sigma_{id}\right)\times\left(\int_{-\infty}^{x}\phi(y\mid\frac{1}{2}(1-S_{id})\times S_s-\theta_{id},\sigma_{id})\,dy\right)dx$
	1	2	$\int_{\theta_{v01}}^{\theta_{v12}} \phi\left(x\mid\frac{1}{2}(S_{id}-1)\times S_s+\theta_{id},\sigma_{id}\right)\times\left(\int_{-\infty}^{x}\phi(y\mid\frac{1}{2}(1-S_{id})\times S_s-\theta_{id},\sigma_{id})\,dy\right)dx$
	1	5	$\int_{\theta_{v14}}^{\infty} \phi\left(x\mid\frac{1}{2}(S_{id}-1)\times S_s+\theta_{id},\sigma_{id}\right)\times\left(\int_{-\infty}^{x}\phi(y\mid\frac{1}{2}(1-S_{id})\times S_s-\theta_{id},\sigma_{id})\,dy\right)dx$
2D Bayesian model	Define: $g(\delta_{id-},\delta_{id+}) \coloneqq P(S_{id}=1\mid\delta_{id-},\delta_{id+}) = \frac{\sum_{j=1}^{5}\phi(\delta_{id+}\mid\Delta t,s)\times\phi(\delta_{id-}\mid 0,s)}{\sum_{j=1}^{5}\left(\phi(\delta_{id+}\mid\Delta t,s)\times\phi(\delta_{id-}\mid 0,s)+\phi(\delta_{id+}\mid 0,s)\times\phi(\delta_{id-}\mid\Delta t,s)\right)}$ and $g^{-1}(\theta,\delta_{id+})$ as the inverse of g as a function of δ_{id-} for fixed δ_{id+}.		
	-1	1	$\frac{\lambda}{10}+(1-\lambda)\times\int_{\mathbb{R}}\phi\left(x\middle\vert\frac{1}{2}\times\Delta t\times(1+S_{id}),s\right)\times\left(\int_{g^{-1}(\theta_{id},x)}^{g^{-1}(\theta_{01},x)}\phi\left(y\middle\vert\frac{1}{2}\times\Delta t\times(1-S_{id}),s\right)dy\right)dx$

	-1	2	$\frac{\lambda}{10}+(1-\lambda)\times\int_{\mathbb{R}}\phi\left(x\middle\|\frac{1}{2}\times\Delta t\times(1+S_{id}),s\right)\times\left(\int_{g^{-1}(\theta_{01},x)}^{g^{-1}(\theta_{02},x)}\phi\left(y\middle\|\frac{1}{2}\times\Delta t\times(1-S_{id}),s\right)dy\right)dx$
	-1	5	$\frac{\lambda}{10}+(1-\lambda)\times\int_{\mathbb{R}}\phi\left(x\middle\|\frac{1}{2}\times\Delta t\times(1+S_{id}),s\right)\times\left(\int_{g^{-1}(\theta_{04},x)}^{\infty}\phi\left(y\middle\|\frac{1}{2}\times\Delta t\times(1-S_{id}),s\right)dy\right)dx$
	1	1	$\frac{\lambda}{10}+(1-\lambda)\times\int_{\mathbb{R}}\phi\left(x\middle\|\frac{1}{2}\times\Delta t\times(1+S_{id}),s\right)\times\left(\int_{g^{-1}(\theta_{11},x)}^{g^{-1}(\theta_{id},x)}\phi\left(y\middle\|\frac{1}{2}\times\Delta t\times(1-S_{id}),s\right)dy\right)dx$
	1	2	$\frac{\lambda}{10}+(1-\lambda)\times\int_{\mathbb{R}}\phi\left(x\middle\|\frac{1}{2}\times\Delta t\times(1+S_{id}),s\right)\times\left(\int_{g^{-1}(\theta_{12},x)}^{g^{-1}(\theta_{11},x)}\phi\left(y\middle\|\frac{1}{2}\times\Delta t\times(1-S_{id}),s\right)dy\right)dx$
	1	5	$\frac{\lambda}{10}+(1-\lambda)\times\int_{\mathbb{R}}\phi\left(x\middle\|\frac{1}{2}\times\Delta t\times(1+S_{id}),s\right)\times\left(\int_{-\infty}^{g^{-1}(\theta_{14},x)}\phi\left(y\middle\|\frac{1}{2}\times\Delta t\times(1-S_{id}),s\right)dy\right)dx$

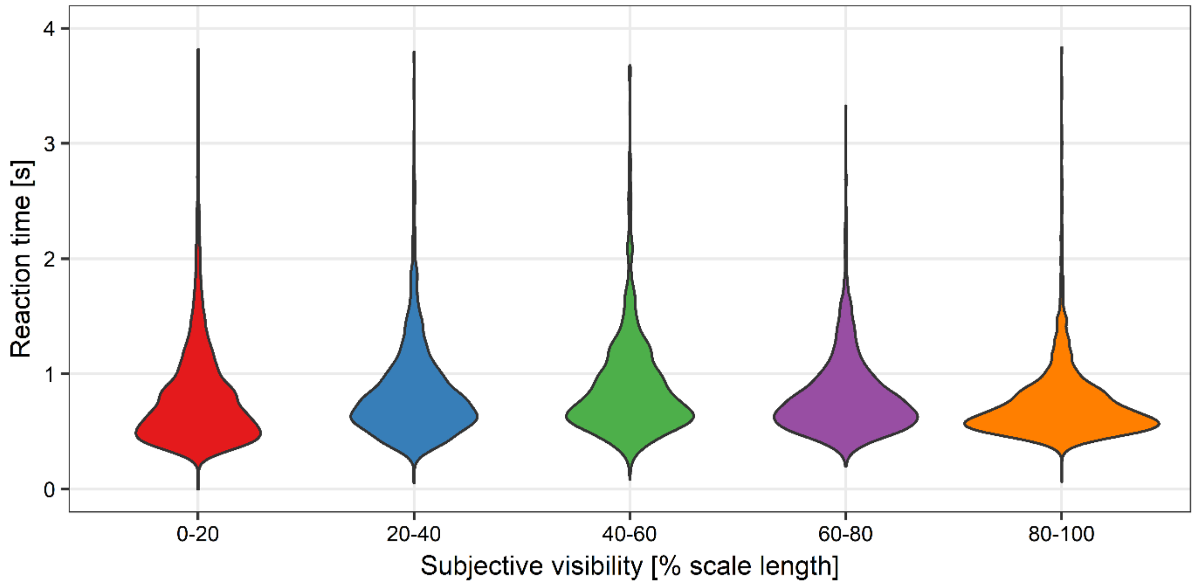

Supplementary Fig S1. Reaction time of the identification judgment in Exp.1 as a function visibility. A Bayesian linear mixed-model regression analysis was neither conclusive about a linear relationship between visibility and reaction times, $BF_{10} = 1.4$, nor about a quadratic relationship, $BF_{10} = 2.4$. The average gamma correlation between visibility and reaction time M_{Γ} was .00, 95% HDI = [-.08 .07], $BF_{10} = .14$.

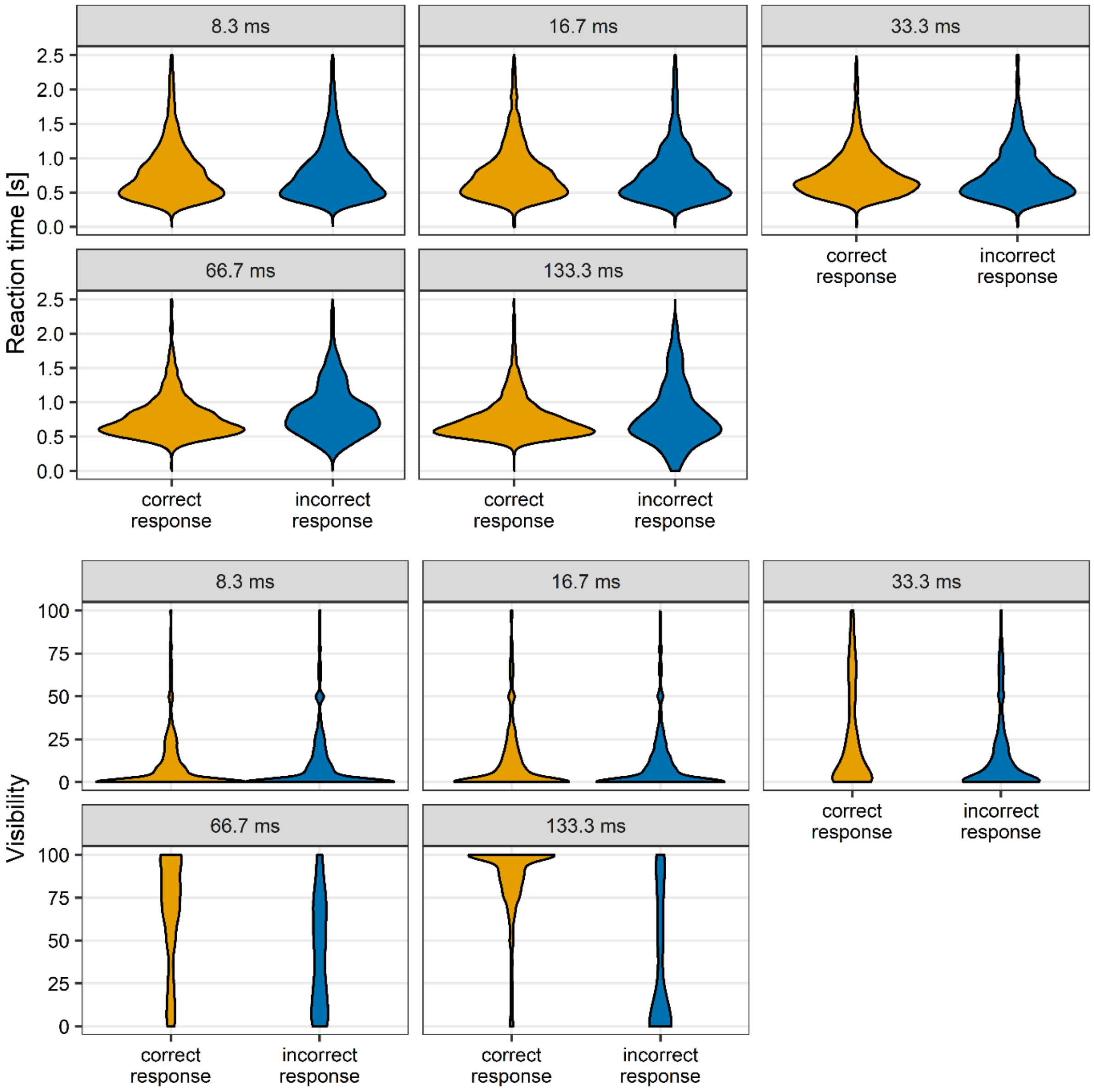

Supplementary Fig S2. Distribution of reaction time (upper two rows) and visibility (lower two rows) as a function of SOA and accuracy of the orientation response (colors). A Bayesian linear mixed-model regression analysis was neither conclusive about an effect of SOA on reaction times in correct trials, $BF_{10} = 1.2$, nor in incorrect trials, $BF_{10} = 0.6$. In contrast, there was extremely strong evidence that visibility increased with SOA in both correct trials, BF_{10}'s = 1.1×10^{55} and in incorrect trials, BF_{10}'s = 1.3×10^{15}.

Supplementary Table S2. Summary statistics of the parameters of the WEV-model obtained during model fitting

Parameter	Experiment 1		Experiment 2 Visibility		Experiment 2 Confidence	
	M	*SEM*	*M*	*SEM*	*M*	*SEM*
S_{S1}	-0.05	0.04	-0.05	0.05	-0.07	0.04
S_{S2}	0.19	0.07	0.06	0.06	0.06	0.06
S_{S3}	0.93	0.15	0.80	0.13	0.82	0.13
S_{S4}	2.91	0.23	2.70	0.19	2.76	0.21
S_{S5}	4.30	0.29	4.25	0.27	4.26	0.25
θ_{id}	0.19	0.07	0.09	0.08	0.09	0.08
w	0.54	0.04	0.59	0.04	0.51	0.03
σ	0.61	0.10	0.58	0.05	0.57	0.04
θ_{v01}	-0.48	0.13	-0.39	0.12	-0.37	0.12
θ_{v02}	-0.98	0.14	-0.87	0.13	-0.79	0.11
θ_{v03}	-1.36	0.17	-1.39	0.13	-1.25	0.12
θ_{v04}	-2.04	0.19	-2.10	0.16	-1.89	0.17
θ_{v11}	0.25	0.22	0.36	0.13	0.38	0.13
θ_{v12}	0.69	0.20	0.83	0.12	0.81	0.12
θ_{v13}	1.08	0.19	1.32	0.12	1.21	0.11
θ_{v14}	1.78	0.16	2.01	0.15	1.79	0.15

Supplementary Table S3. Correlation between the WEV-model parameters fitted to visibility and the parameters fitted to confidence in Exp. 2

Parameter	r	95% HDI	
		Lower	Upper
S_{S1}	.92	.82	.95
S_{S2}	.94	.86	.96
S_{S3}	.98	.96	.99
S_{S4}	.98	.94	.98
S_{S5}	.97	.94	.98
θ_{id}	>.99	>.99	>.99
w	.69	.43	.80
σ	.73	.48	.82
θ_{v01}	.58	.28	.73
θ_{v02}	.60	.30	.74
θ_{v03}	.67	.39	.78
θ_{v04}	.66	.39	.78
θ_{v11}	.73	.49	.83
θ_{v12}	.71	.46	.82
θ_{v13}	.55	.23	.70
θ_{v14}	.51	.19	.68

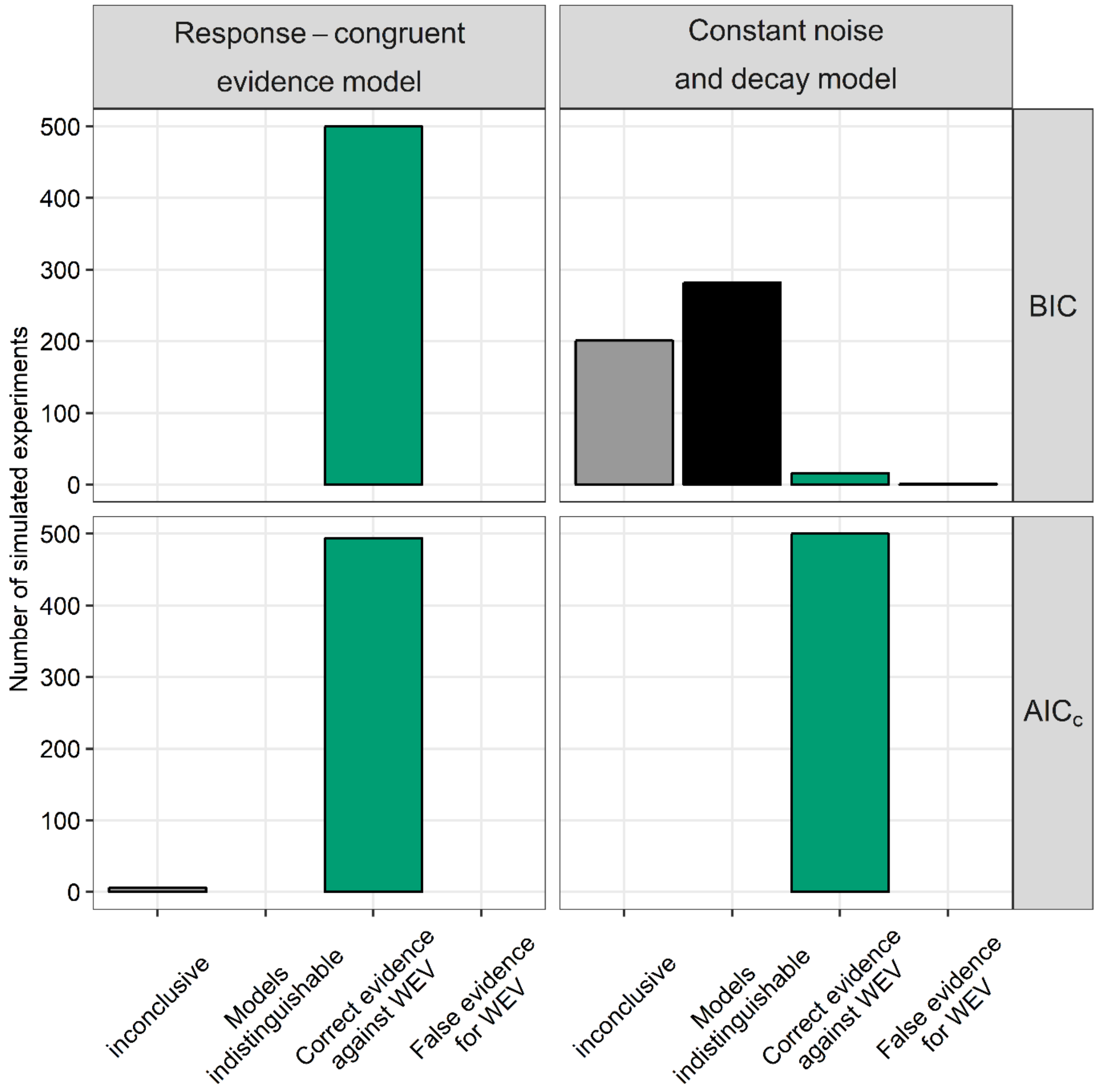

Supplementary Fig S3. Results of comparisons between model fits to simulated data between the true generative model and the WEV model. The true generative models were the response-congruent evidence model for the panels on the left and constant noise and decay model for the panels on the right. The upper and lower panels show the results based on the BIC and AIC_c, respectively. The outcome of the comparison was interpreted as follows:

- "inconclusive" if the Bayes factor fell between 1/3 and 3,
- "indistinguishable" if the Bayes factor was below 1/3
- "Correct evidence against the WEV-model" if the Bayes factor was greater than 3 and if the mean AIC_c/BIC of the generative model were smaller than mean AIC_c/BIC of the WEV-model
- "False evidence against the WEV-model" if the Bayes factor was greater than 3 and if the mean AIC_c/BIC of the WEV-model were smaller than mean AIC_c/BIC of the generative model.

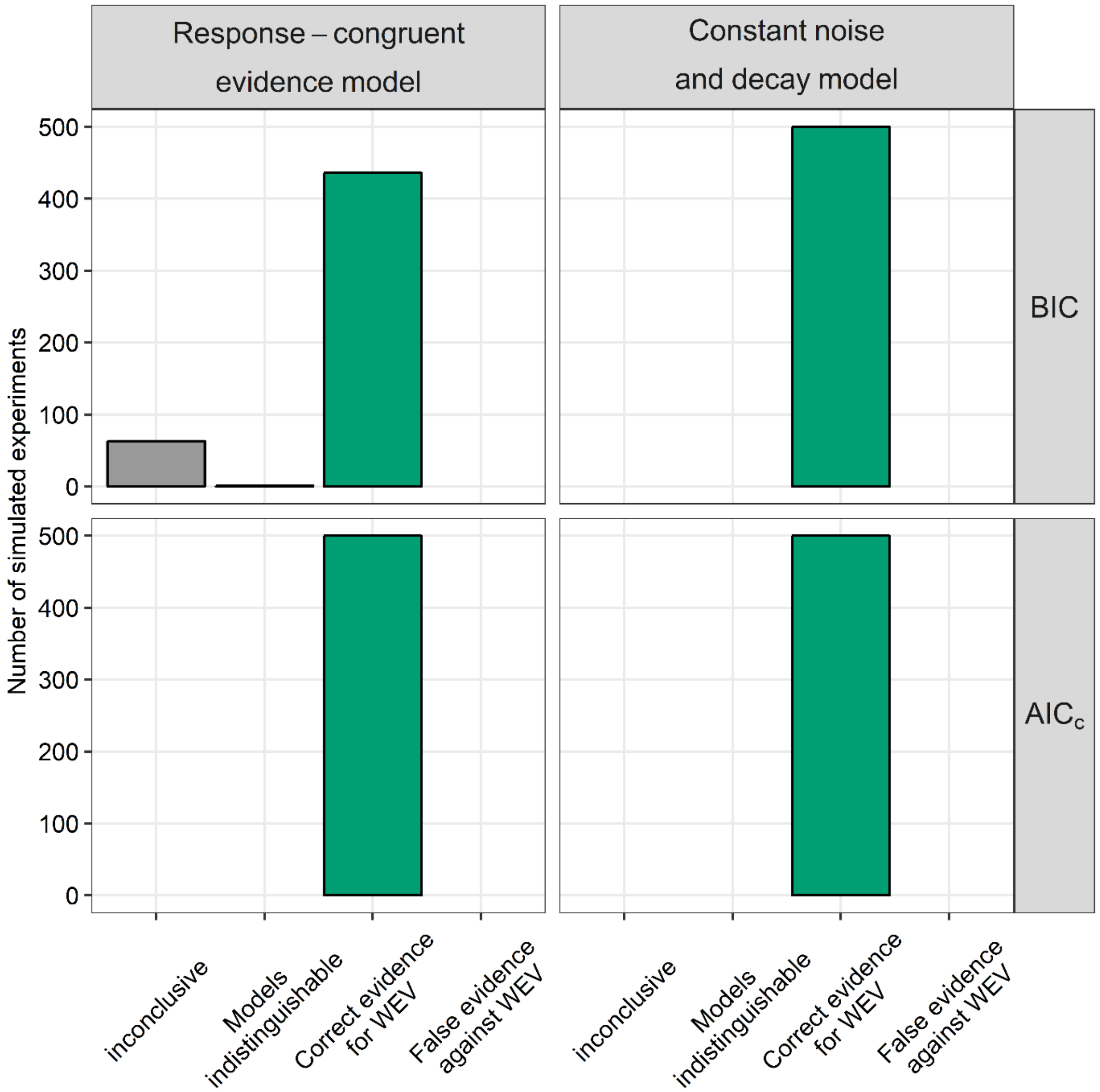

Supplementary Fig S4. Results of comparisons between model fits of WEV model, the response-congruent evidence model, and constant noise and decay model based on data generated according to the WEV model. The upper and lower panels show the results based on BIC and AIC_c, respectively. The outcome of the comparison was interpreted as follows:

- "inconclusive" if the Bayes factor fell between 1/3 and 3,
- "indistinguishable" if the Bayes factor was below 1/3
- "Correct evidence for the WEV-model" if the Bayes factor was greater than 3 and if the mean AIC_c/BIC of the WEV-model were smaller than mean AIC_c/BIC of the other model
- "False evidence against the WEV-model" if the Bayes factor was greater than 3 and if the mean AIC_c/BIC of the WEV-model were greater than mean AIC_c/BIC of the other model.

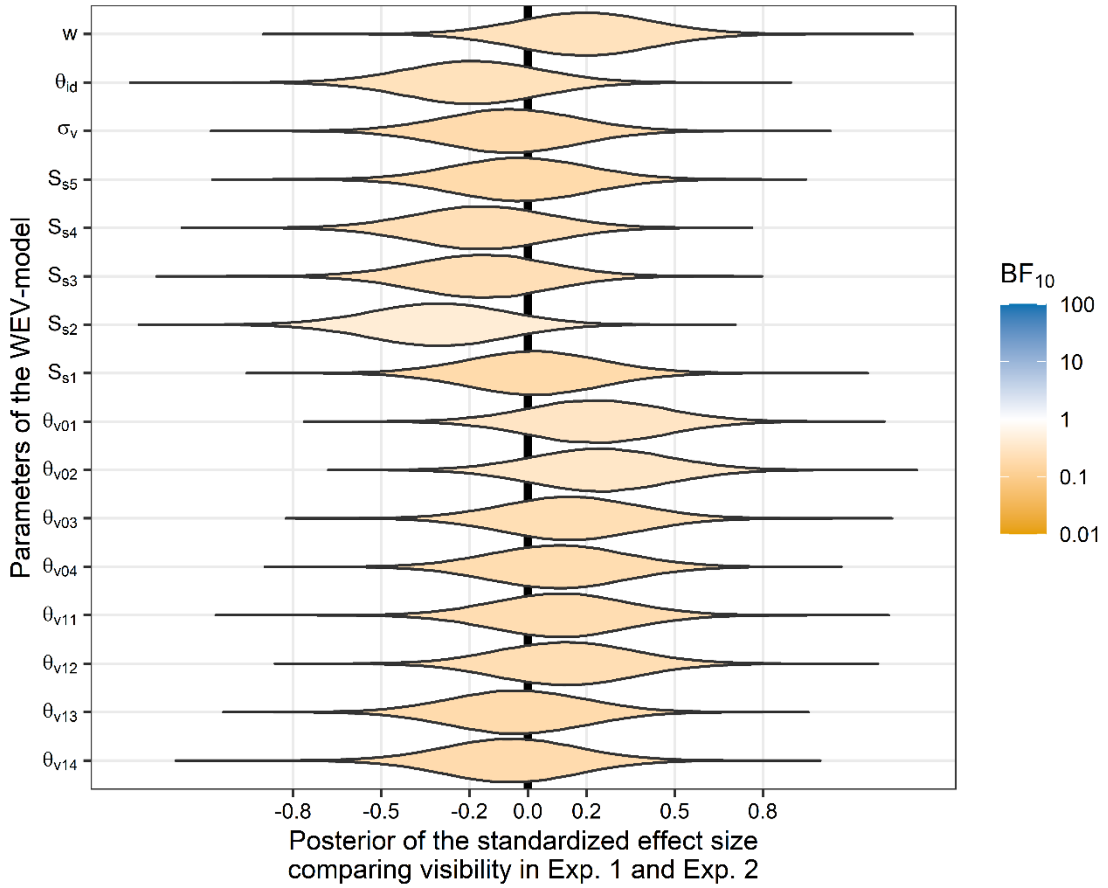

Supplementary Fig S5. Posterior distributions of the standardized mean difference between visibility in Exp. 1 and Exp. 2 with respect to each parameter of the WEV model. Colours indicate the strength of evidence in favour (blue) or against (orange) a difference between the two experiments.

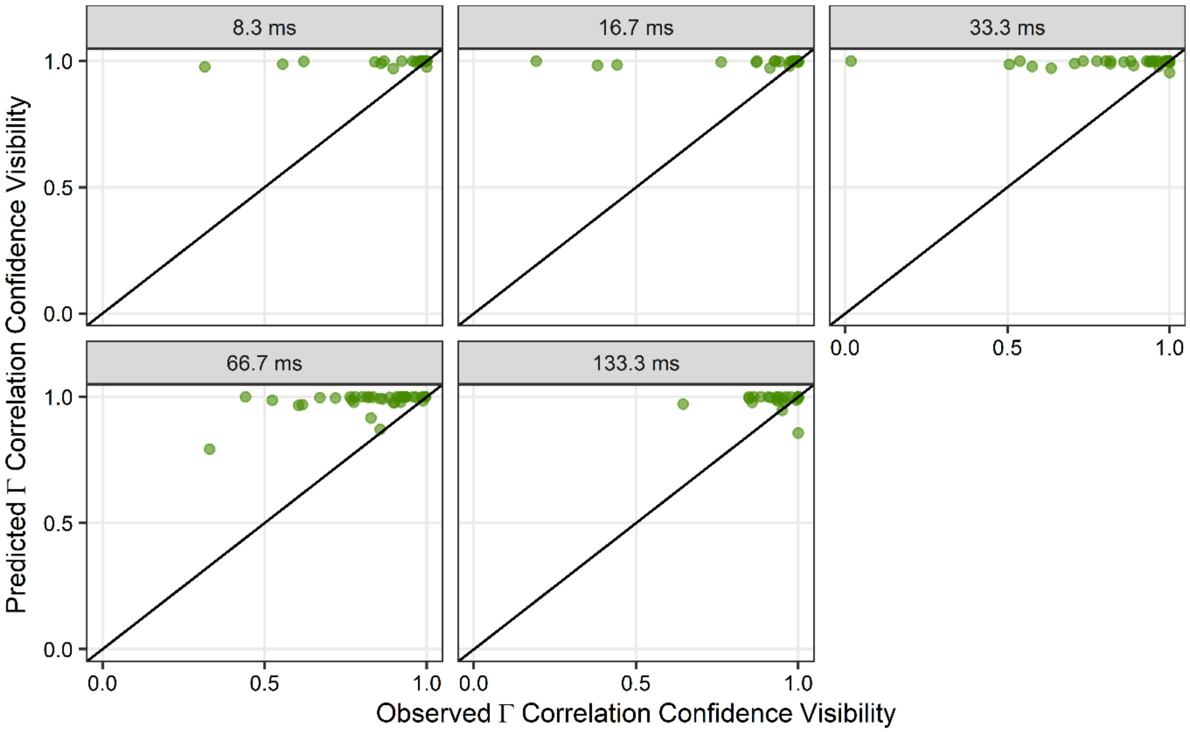

Supplementary Fig S6. Observed gamma correlation coefficients between subjective visibility and decision confidence rating vs. gamma correlation coefficients estimated from the WEV model with the corresponding fitted parameters for different SOA levels (panels). Each point represents data from a participant. Parameters for the random samples were equal to the mean of the fitted parameters for all parameters but the weight w.
